# Supplementary figures and images for: Distinct senotypes in p16- and p21-positive cells across human and mouse aging tissues
Source: EMBO J. 2025 Oct 29;44(23):7295–325. doi: 10.1038/s44318-025-00601-2 (PMC12669595; doi:10.1038/s44318-025-00601-2)

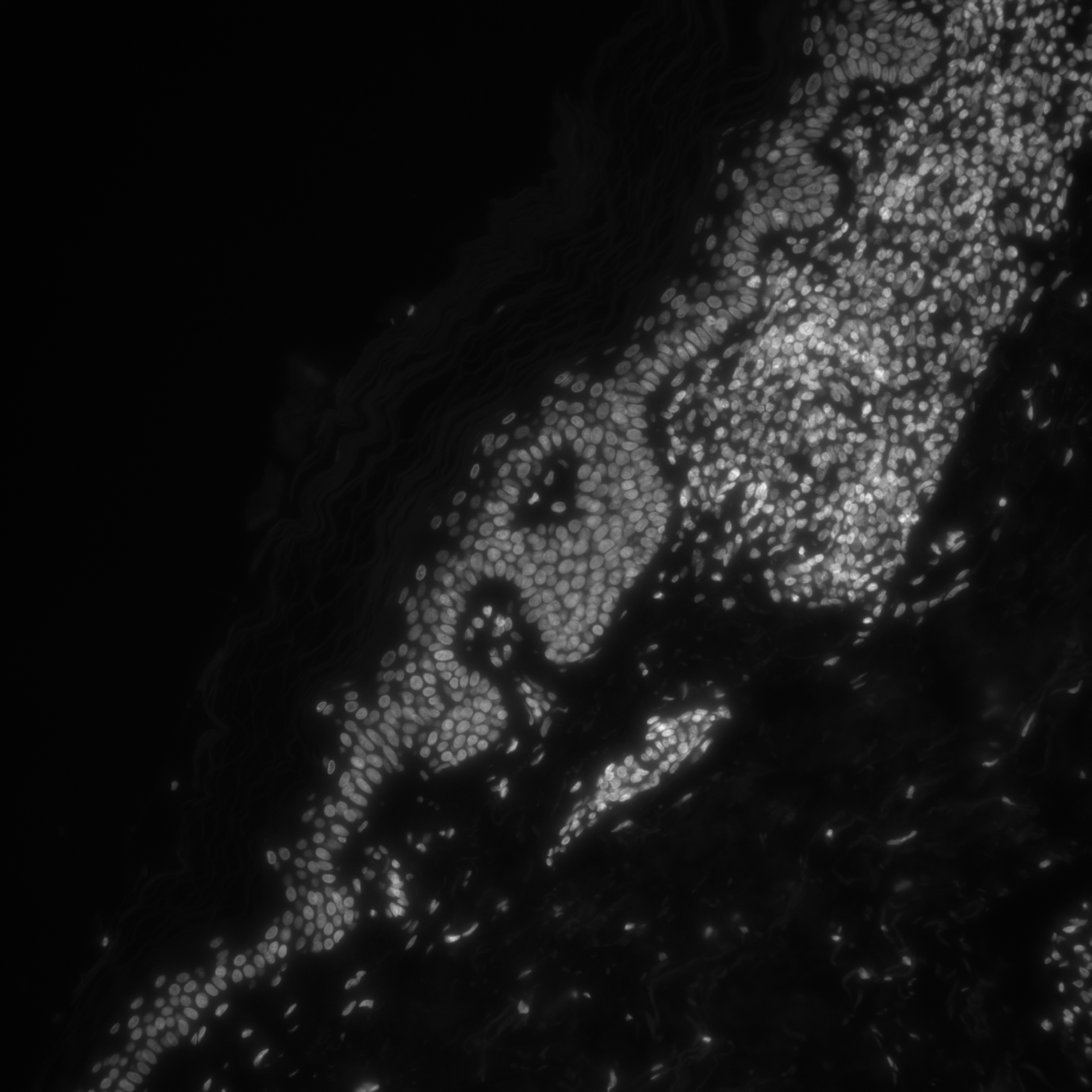

Supplement: Supplementary file 2 — Source data Fig. 3 [file 44318_2025_601_MOESM2_ESM.zip › Figure3_Source_Data/E/left/5138 skin p21 green p16 red 26jun25.lif - DAPI.tif]

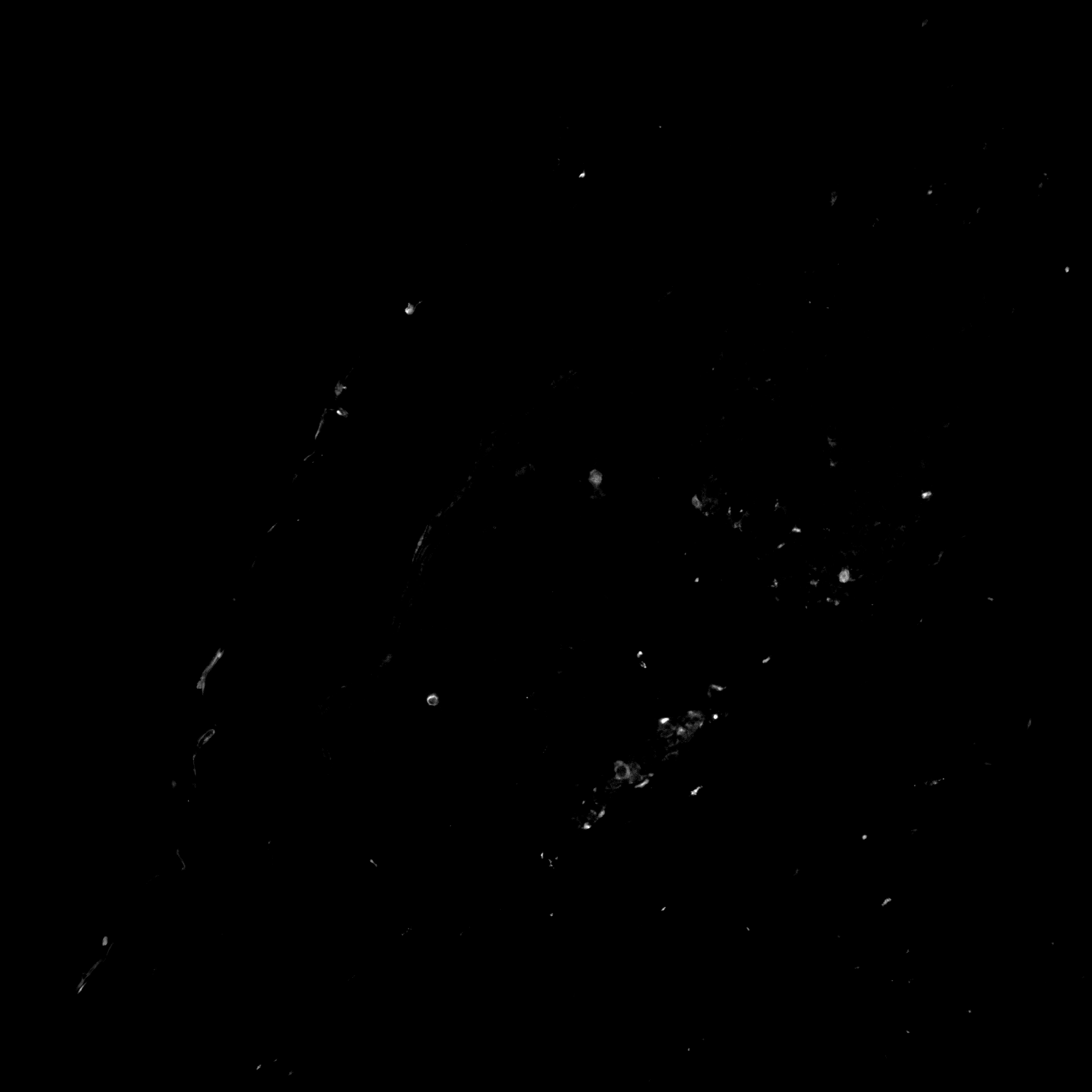

Supplement: Supplementary file 2 — Source data Fig. 3 [file 44318_2025_601_MOESM2_ESM.zip › Figure3_Source_Data/E/left/5138 skin p21 green p16 red 26jun25.lif - p16.tif]

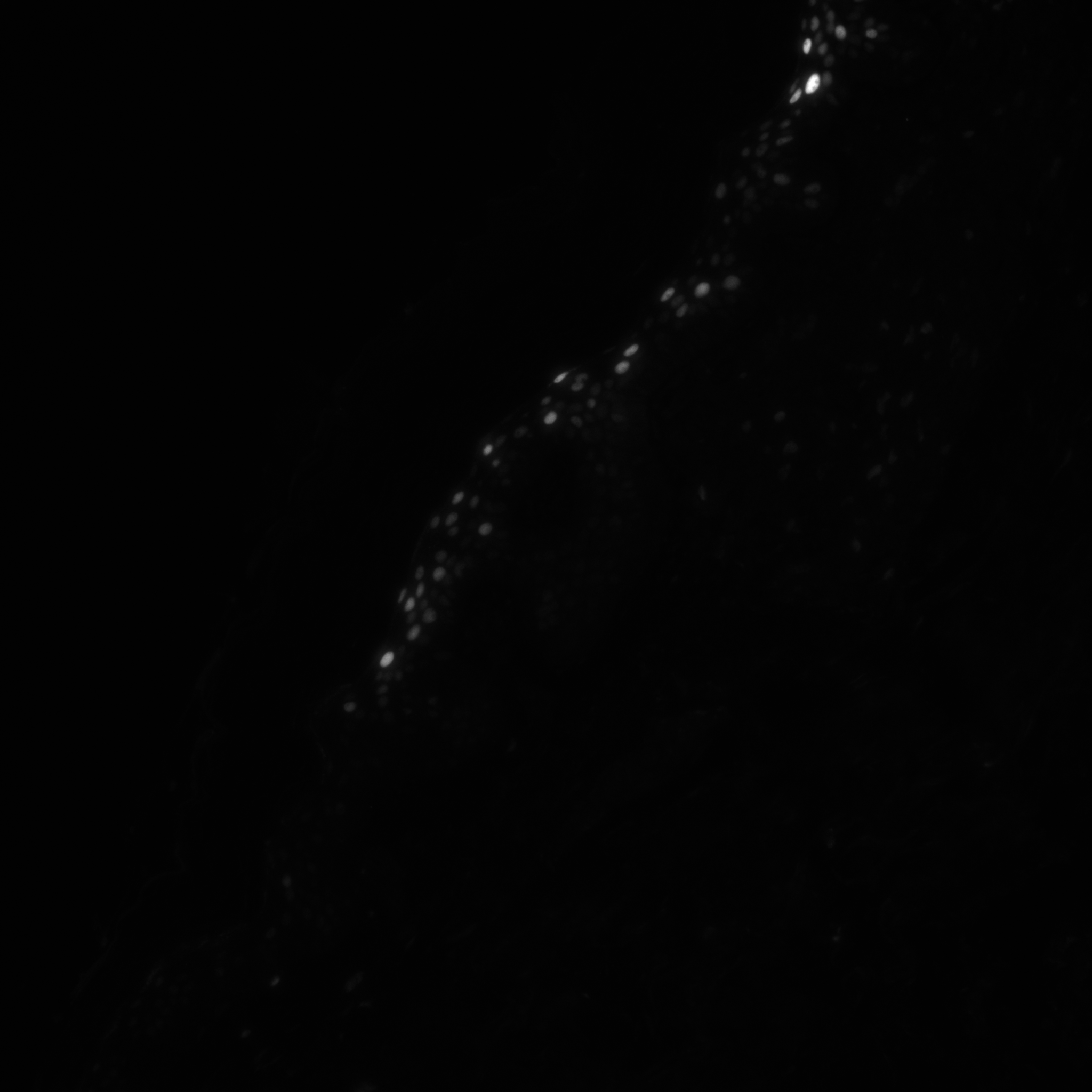

Supplement: Supplementary file 2 — Source data Fig. 3 [file 44318_2025_601_MOESM2_ESM.zip › Figure3_Source_Data/E/left/5138 skin p21 green p16 red 26jun25.lif - p21.tif]

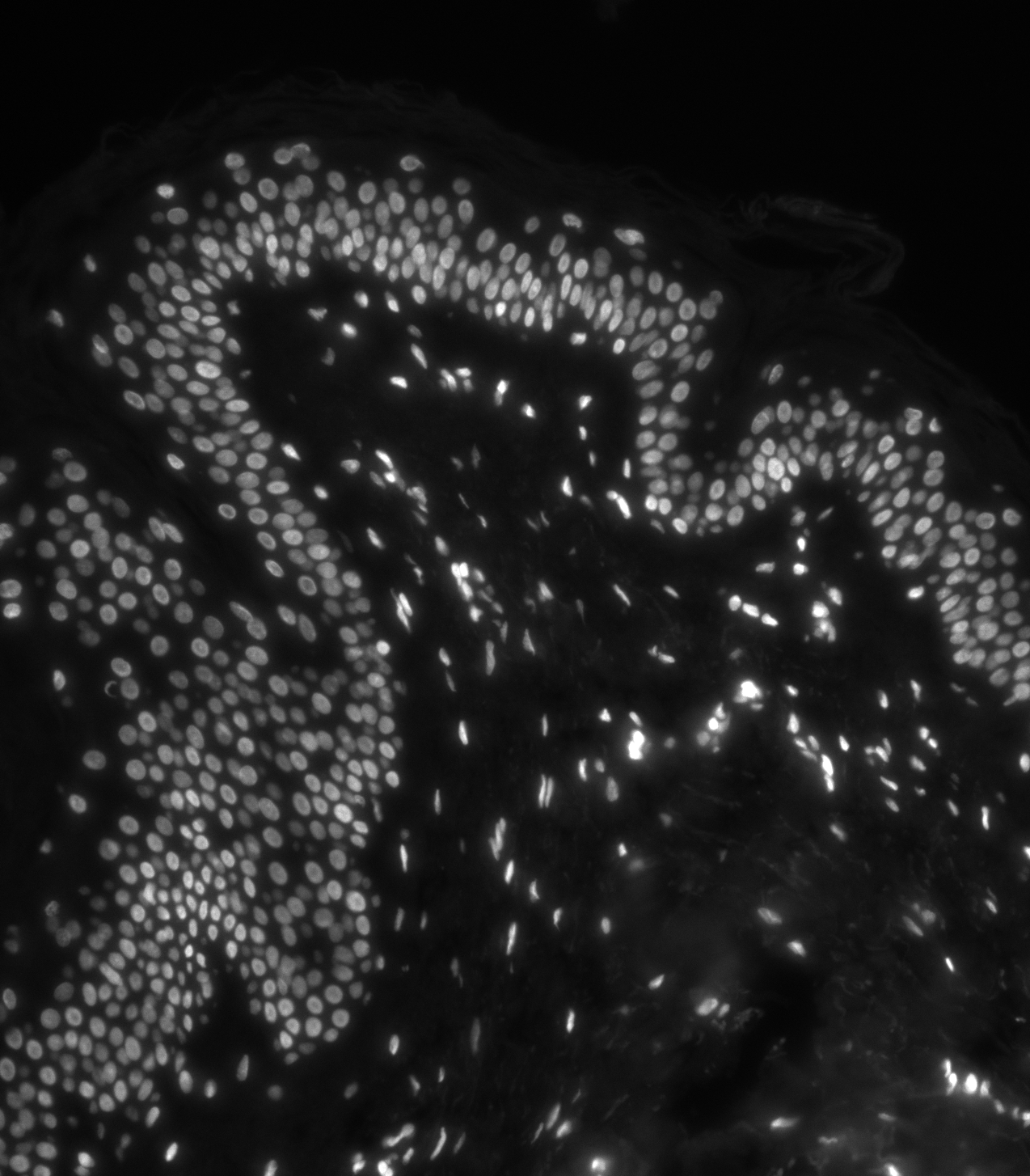

Supplement: Supplementary file 2 — Source data Fig. 3 [file 44318_2025_601_MOESM2_ESM.zip › Figure3_Source_Data/E/mid/5014 skin p21 green p16 red 26jun25.lif - DAPI.tif]

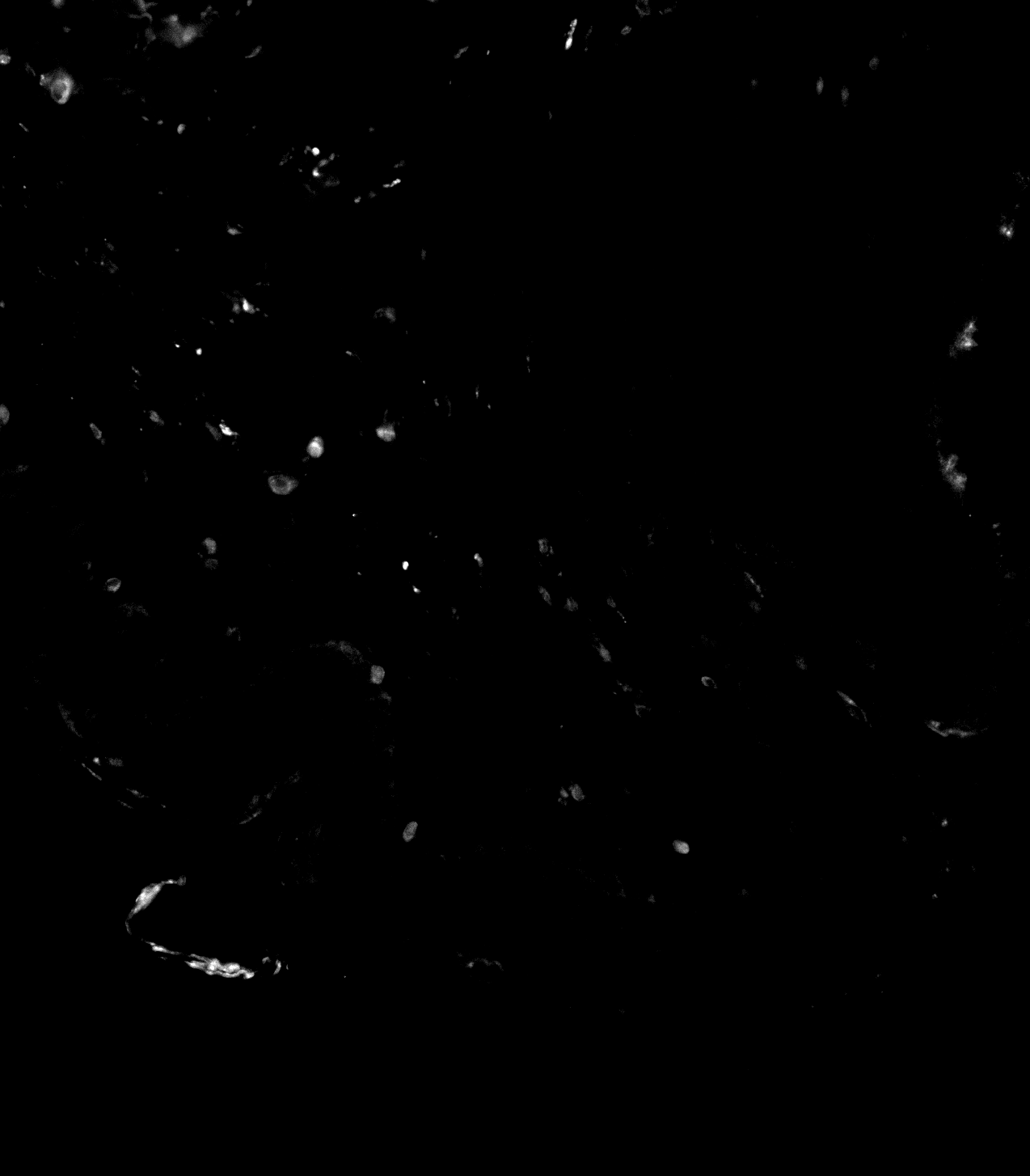

Supplement: Supplementary file 2 — Source data Fig. 3 [file 44318_2025_601_MOESM2_ESM.zip › Figure3_Source_Data/E/mid/5014 skin p21 green p16 red 26jun25.lif - p16.tif]

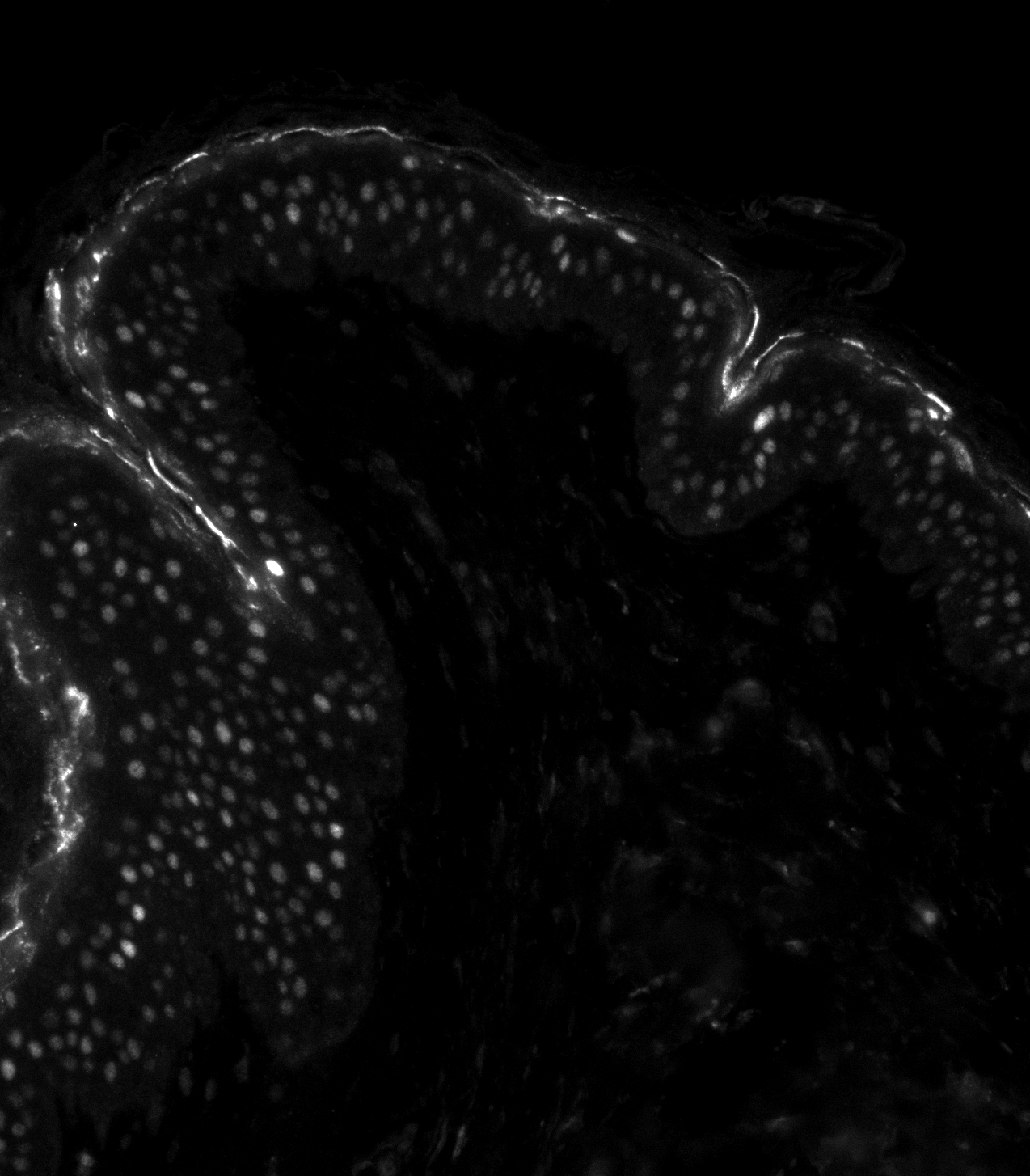

Supplement: Supplementary file 2 — Source data Fig. 3 [file 44318_2025_601_MOESM2_ESM.zip › Figure3_Source_Data/E/mid/5014 skin p21 green p16 red 26jun25.lif - p21.tif]

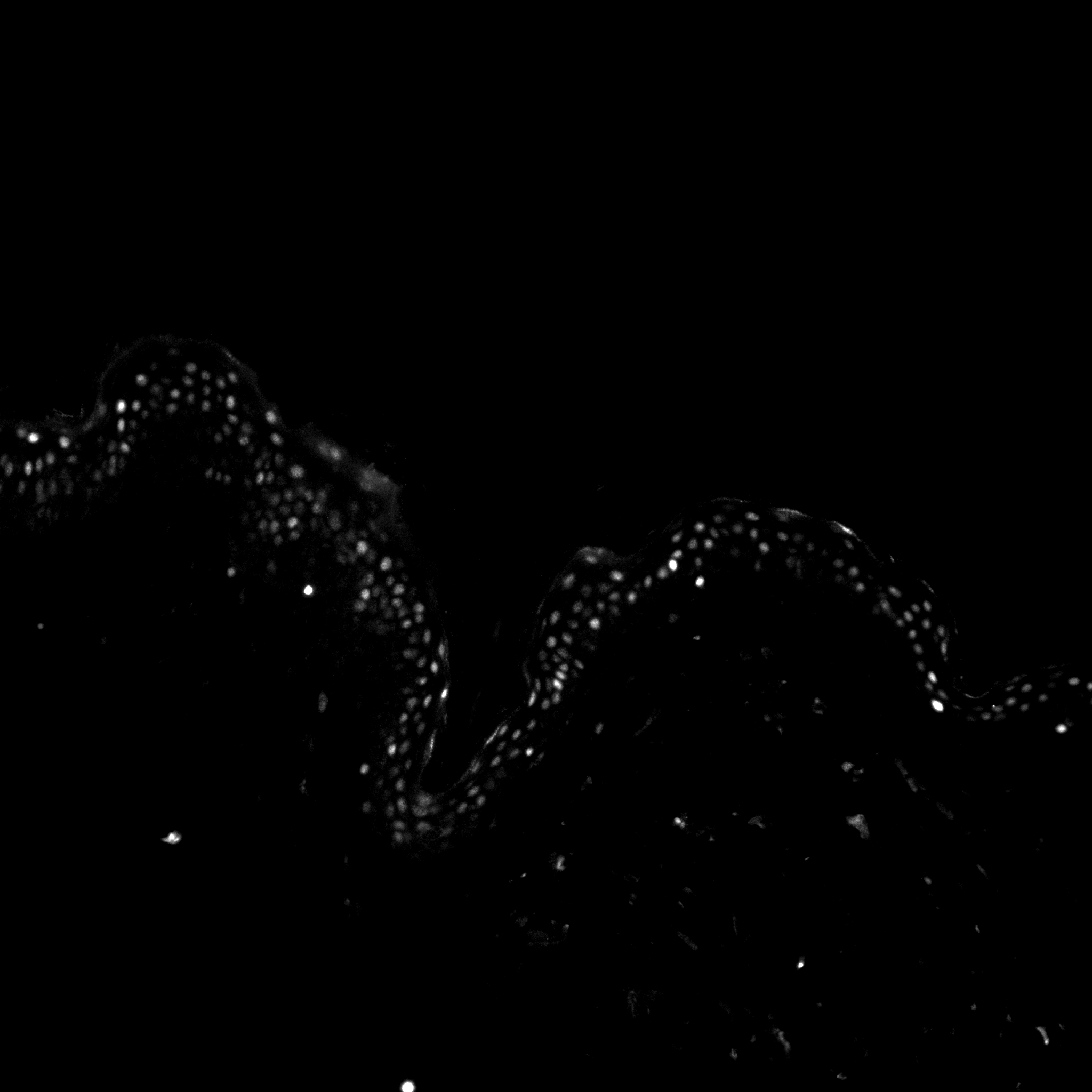

Supplement: Supplementary file 2 — Source data Fig. 3 [file 44318_2025_601_MOESM2_ESM.zip › Figure3_Source_Data/E/right/4891 skin p21 green p16 red 26jun25.lif - p21.tif]

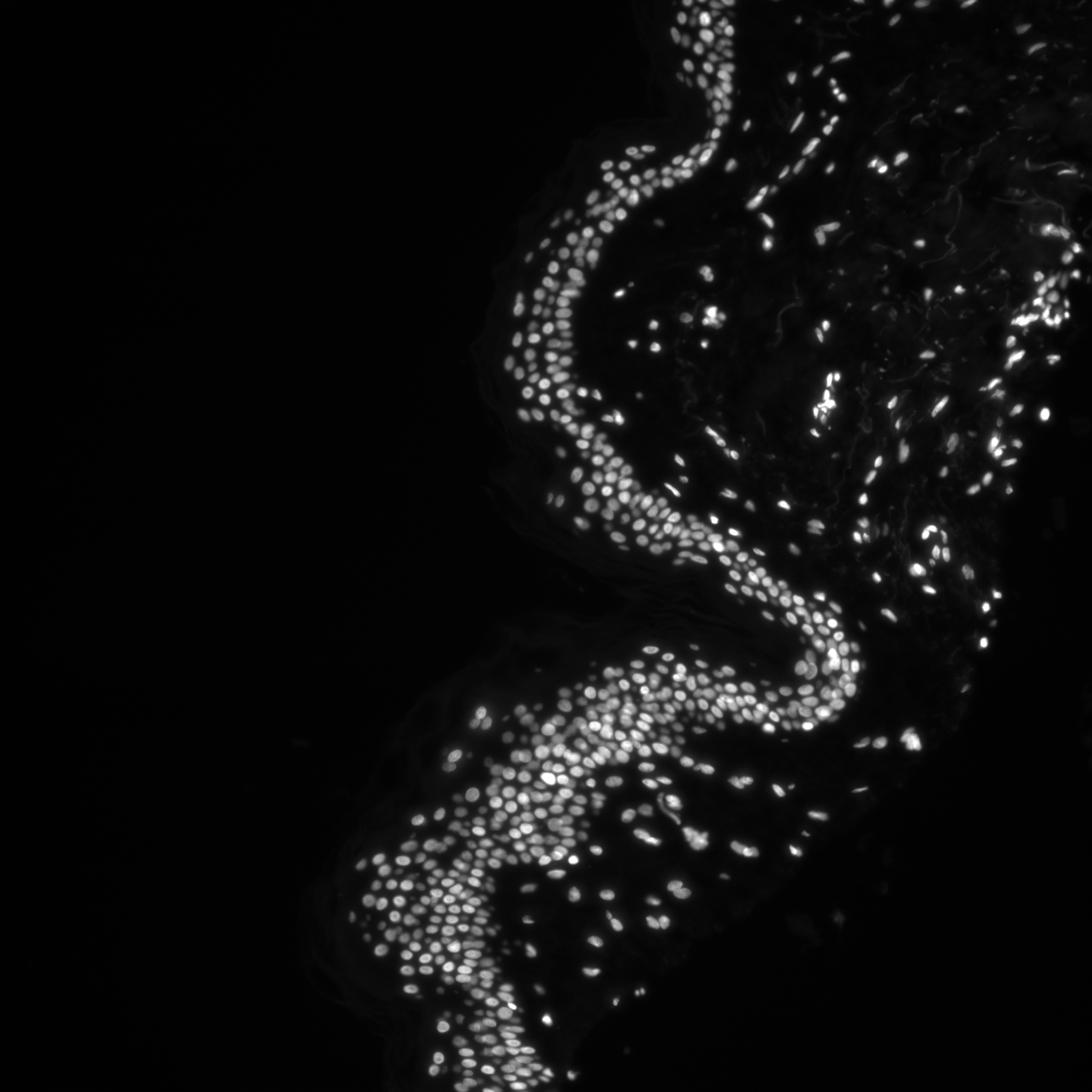

Supplement: Supplementary file 2 — Source data Fig. 3 [file 44318_2025_601_MOESM2_ESM.zip › Figure3_Source_Data/E/right/C1-4891 skin p21 green p16 red 26jun25.lif - DAPI.tif]

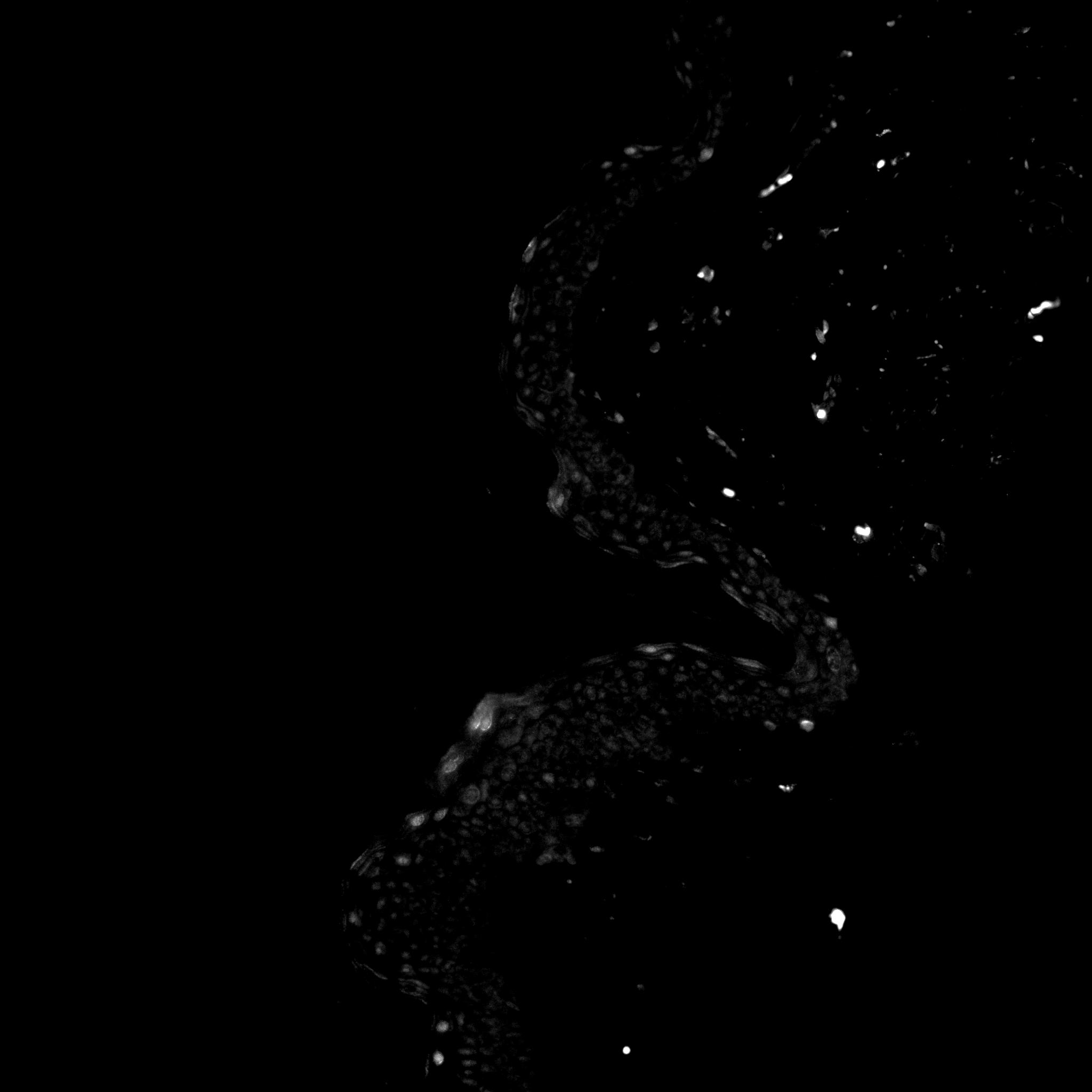

Supplement: Supplementary file 2 — Source data Fig. 3 [file 44318_2025_601_MOESM2_ESM.zip › Figure3_Source_Data/E/right/C2-4891 skin p21 green p16 red 26jun25.lif - p16.tif]

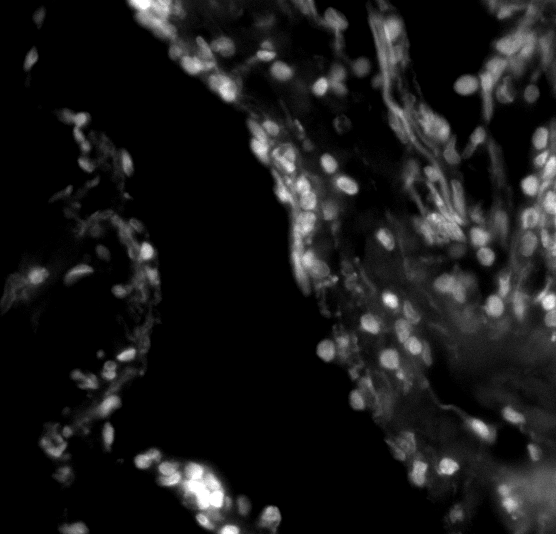

Supplement: Supplementary file 2 — Source data Fig. 3 [file 44318_2025_601_MOESM2_ESM.zip › Figure3_Source_Data/J/left/1626-2 COPD lung p21 green p16 red 26jun25.lif - DAPI .tif]

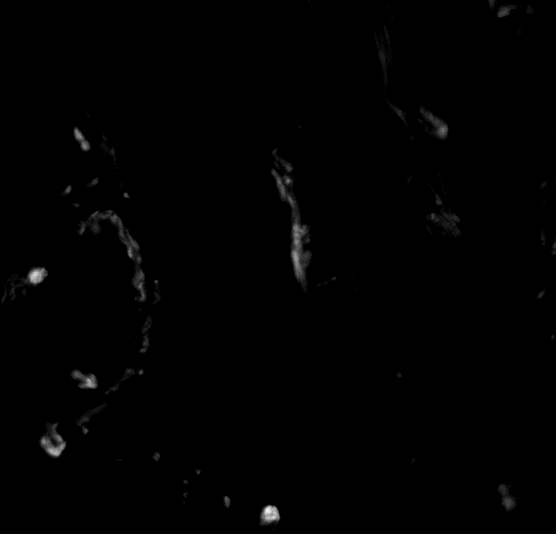

Supplement: Supplementary file 2 — Source data Fig. 3 [file 44318_2025_601_MOESM2_ESM.zip › Figure3_Source_Data/J/left/1626-2 COPD lung p21 green p16 red 26jun25.lif - p16 .tif]

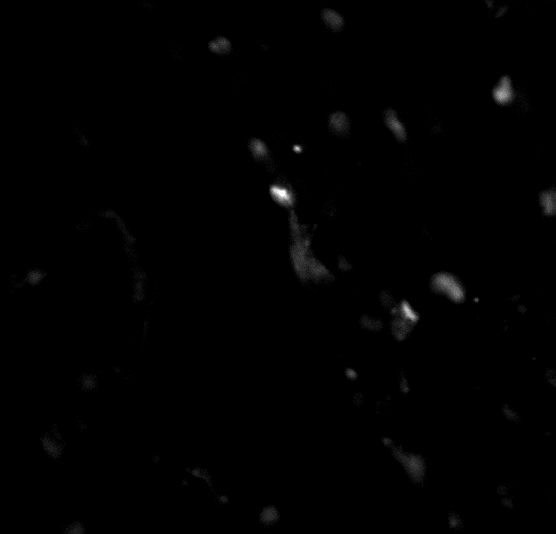

Supplement: Supplementary file 2 — Source data Fig. 3 [file 44318_2025_601_MOESM2_ESM.zip › Figure3_Source_Data/J/left/1626-2 COPD lung p21 green p16 red 26jun25.lif - p21 .tif]

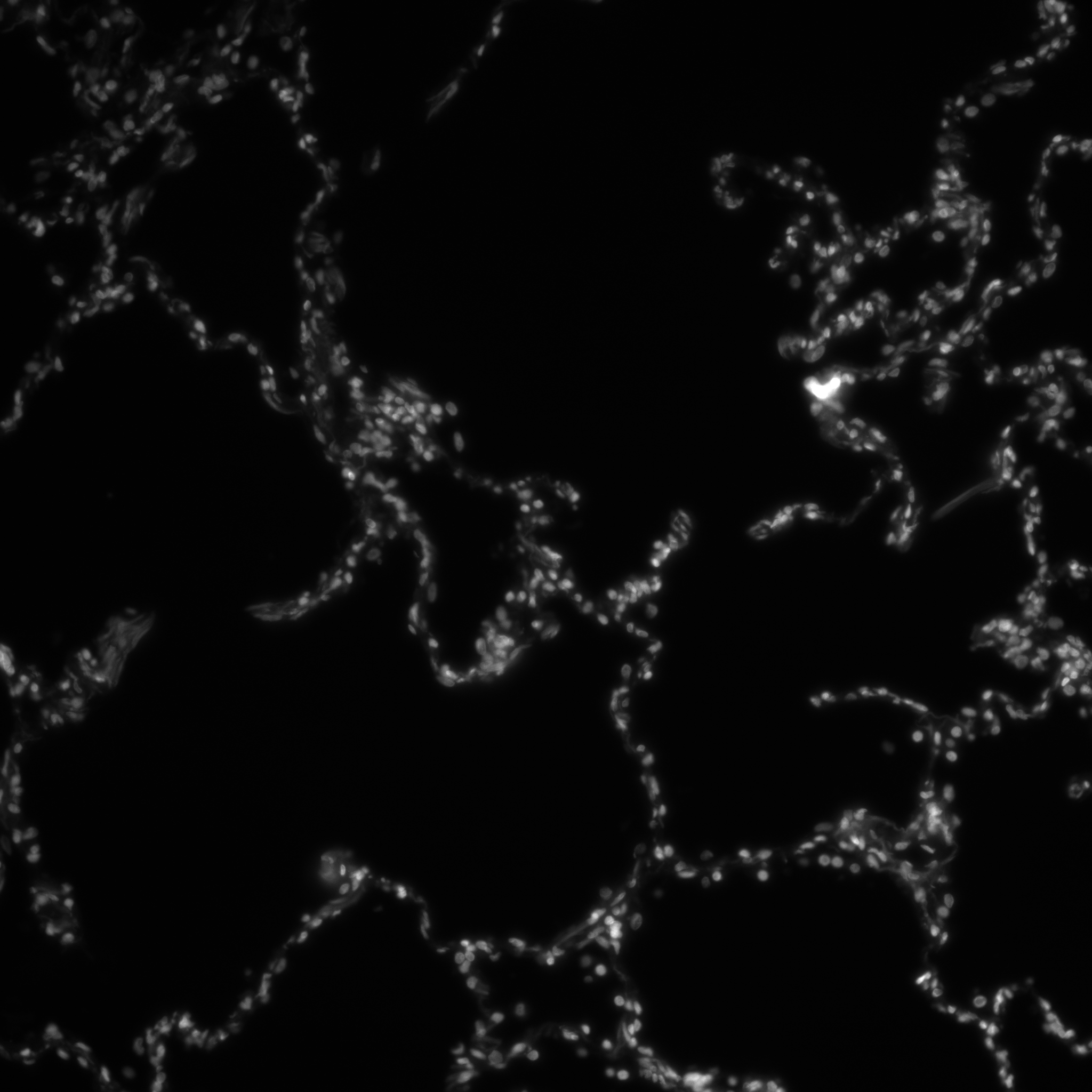

Supplement: Supplementary file 2 — Source data Fig. 3 [file 44318_2025_601_MOESM2_ESM.zip › Figure3_Source_Data/J/mid/1627-1 lung IPF p21 green p16 red 26jun25_Image 5_DAPI.tif]

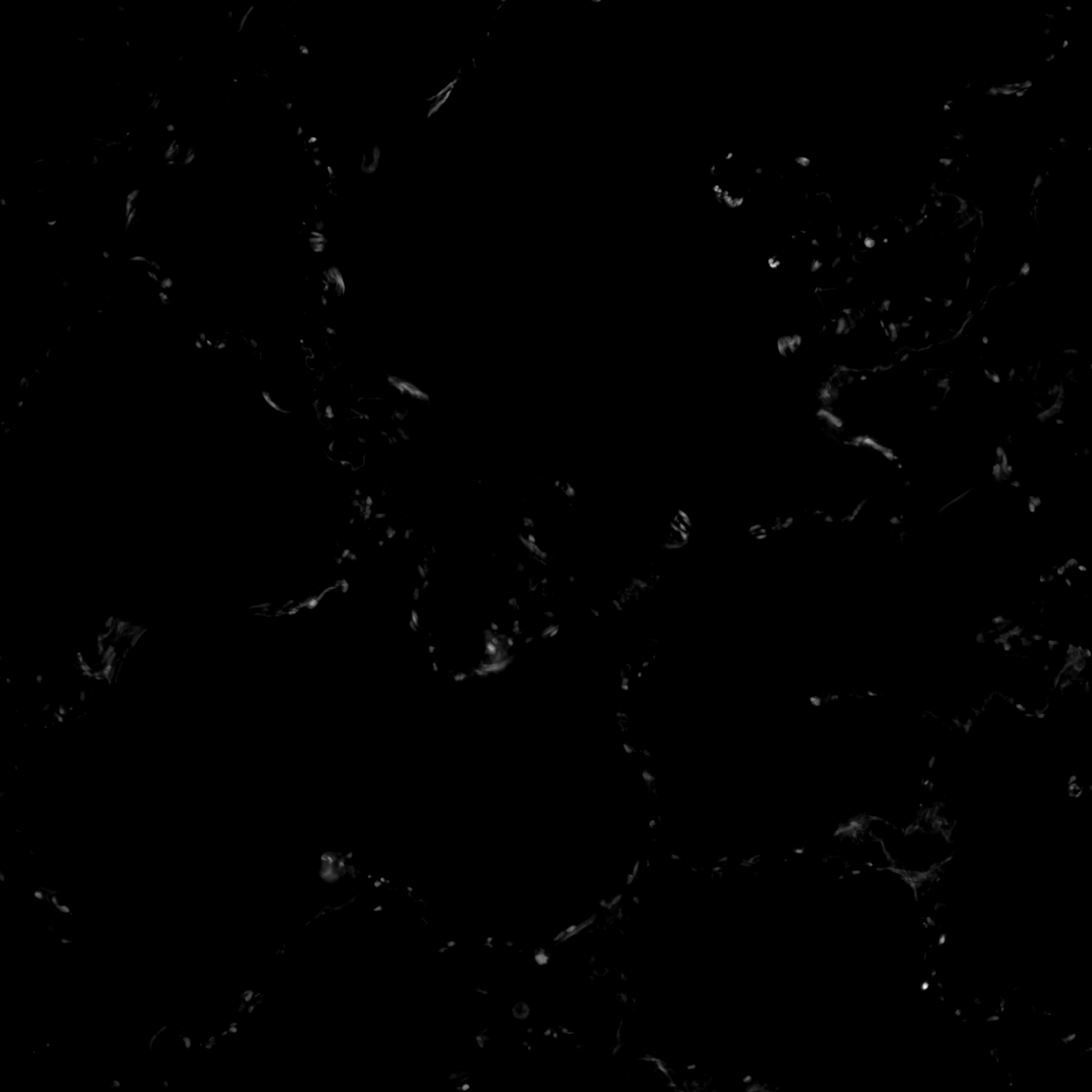

Supplement: Supplementary file 2 — Source data Fig. 3 [file 44318_2025_601_MOESM2_ESM.zip › Figure3_Source_Data/J/mid/1627-1 lung IPF p21 green p16 red 26jun25_Image 5_p16.tif]

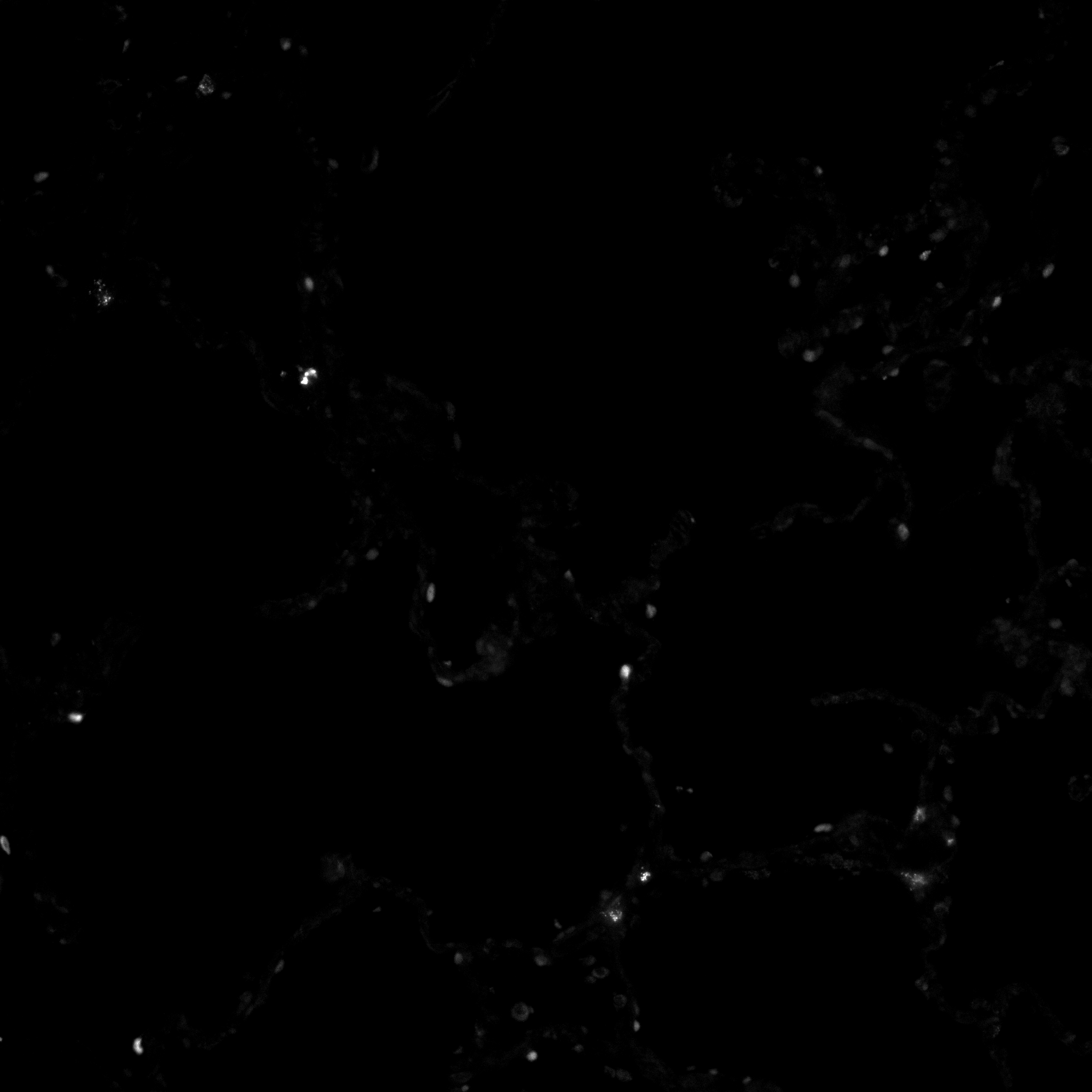

Supplement: Supplementary file 2 — Source data Fig. 3 [file 44318_2025_601_MOESM2_ESM.zip › Figure3_Source_Data/J/mid/1627-1 lung IPF p21 green p16 red 26jun25_Image 5_p21.tif]

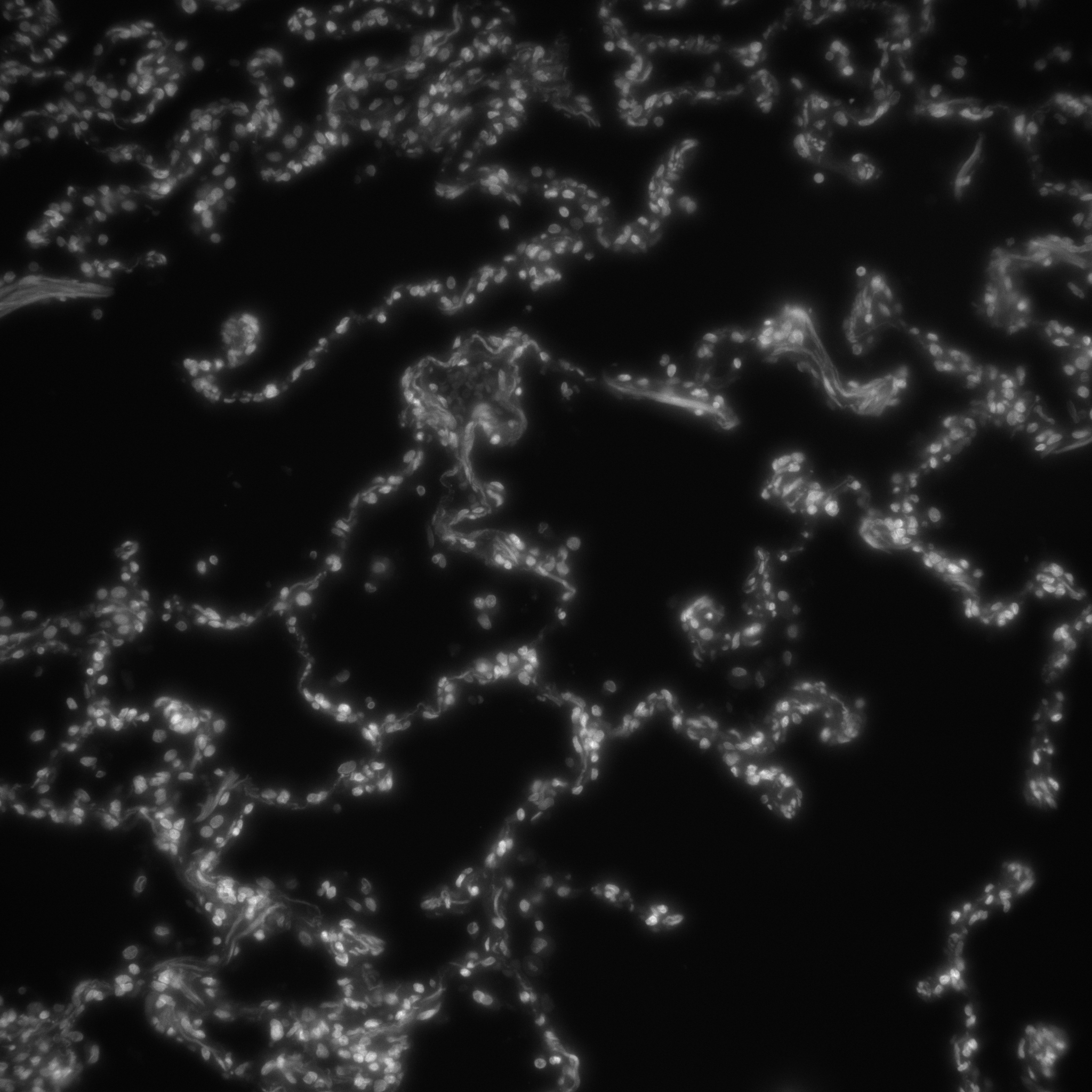

Supplement: Supplementary file 2 — Source data Fig. 3 [file 44318_2025_601_MOESM2_ESM.zip › Figure3_Source_Data/J/right/1627-1 lung IPF p21 green p16 red 26jun25_Image 8_DAPI.tif]

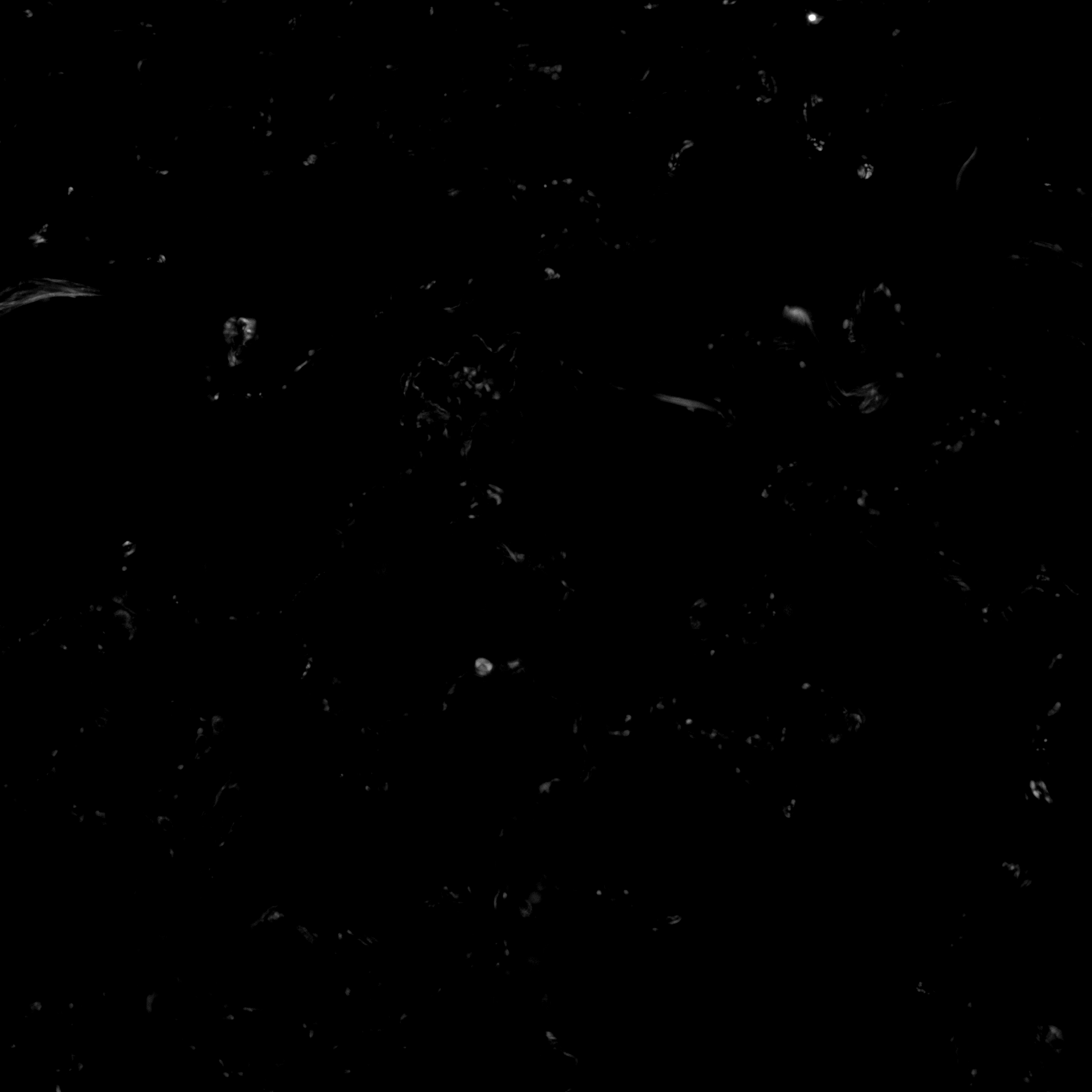

Supplement: Supplementary file 2 — Source data Fig. 3 [file 44318_2025_601_MOESM2_ESM.zip › Figure3_Source_Data/J/right/1627-1 lung IPF p21 green p16 red 26jun25_Image 8_p16.tif]

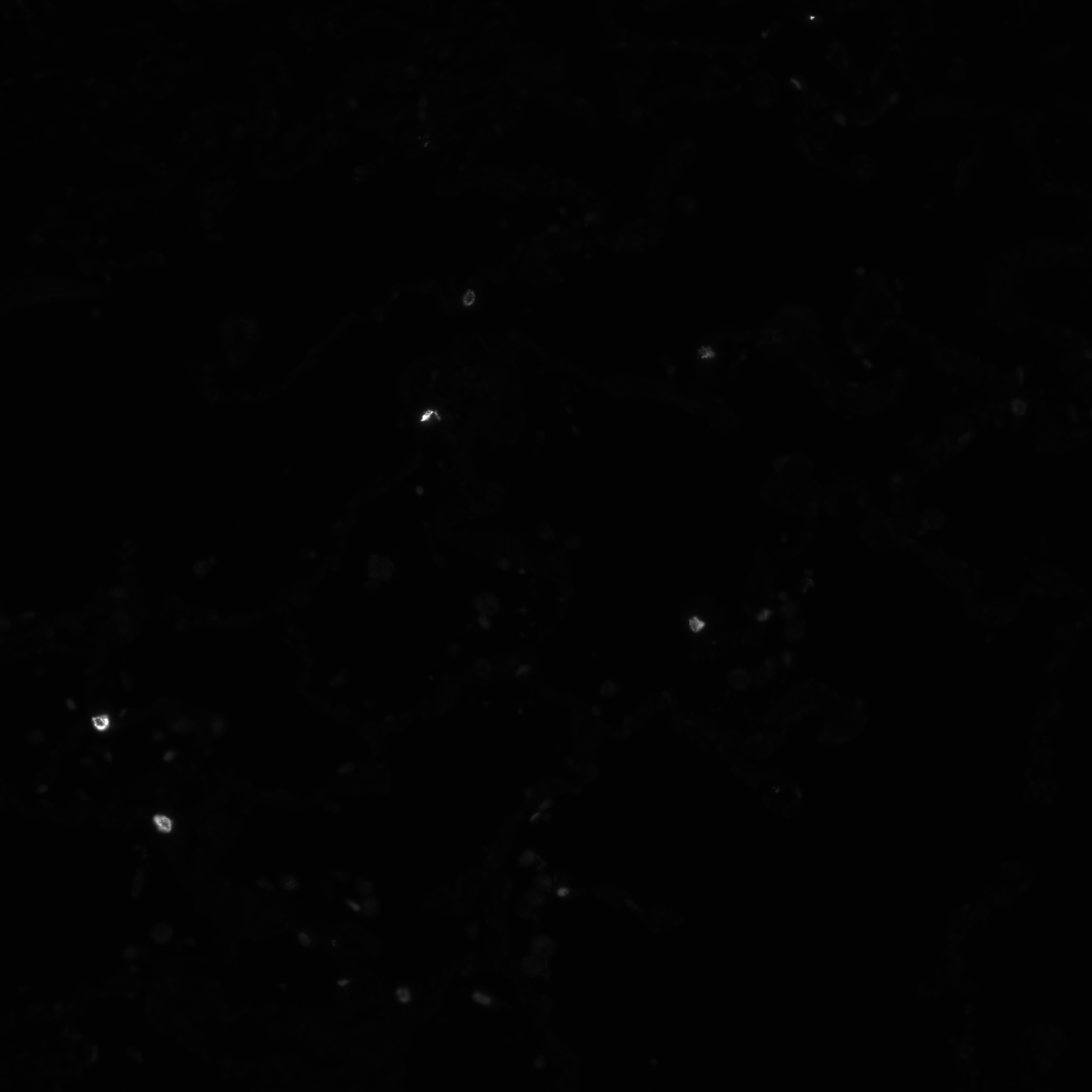

Supplement: Supplementary file 2 — Source data Fig. 3 [file 44318_2025_601_MOESM2_ESM.zip › Figure3_Source_Data/J/right/1627-1 lung IPF p21 green p16 red 26jun25_Image 8_p21.tif]
