# Supplementary material for: Distinct senotypes in p16- and p21-positive cells across human and mouse aging tissues
Source: EMBO J. 2025 Oct 29;44(23):7295–325. doi: 10.1038/s44318-025-00601-2 (PMC12669595; doi:10.1038/s44318-025-00601-2)
Supplement: Supplementary file 3 — Expanded View Figures [file 44318_2025_601_MOESM3_ESM.pdf]

Expanded View Figures

Figure EV1. Comprehensive examination of the mRNA expression profiles for the whole secretome and SenMayo in aging murine hippocampus.

(**A**) *p16*+ cells are significantly more frequent in the old brain. (**B**) Microglia has more *p21*+ cells in the old brain. (**C**) In the murine hippocampus, there is minimal overlap between the mRNA expression of the whole secretome + SenMayo in *p21*+ and *p16*+ cells. (**D**) SenMayo composition in *p16*+ vs. *p21*+ microglia shows some common secretory genes like *Plaur* and *Cxcl16*, while in (**E**) oligodendrocytes, there is no common secretory phenotype. (**F**) The velocity trajectory of *p16*+ cells, *p21*+ cells and the remaining brain cells shows no overlap or common origin (**G**) Monocle3 applied to the backbone from f shows that there is no common "ancestor" for these cell types. **A**: unpaired *t*-test, $n = 2$ per condition, error bars: sd, **B**: multiple paired *t*-tests, $n = 2$ per condition, error bars: sd.

© The Author(s)

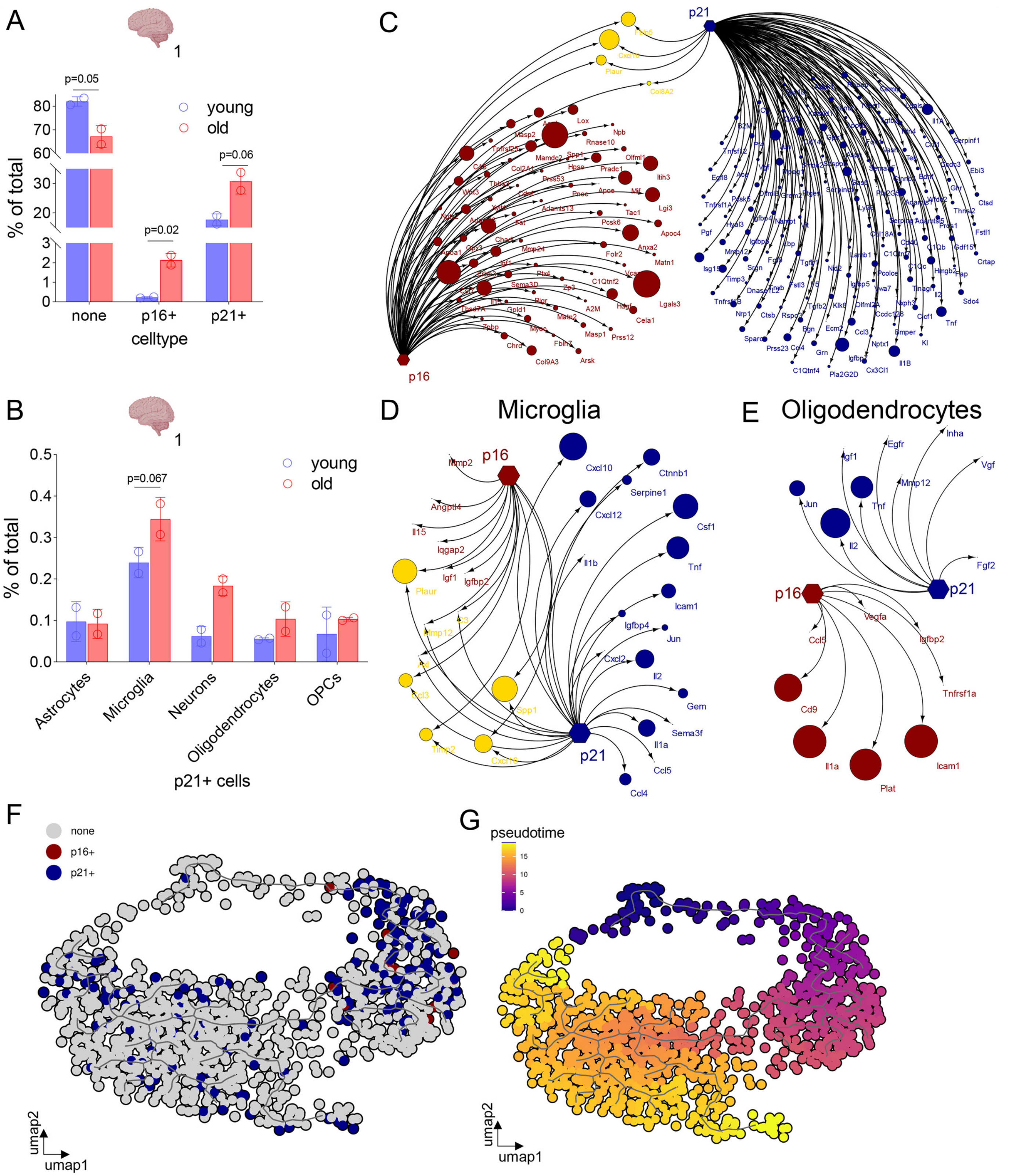
A
1
p=0.05
p=0.02
p=0.06
young
old
% of total
none
p16+
p21+
celltype
C
p21
p16
B
1
p=0.067
young
old
% of total
Astrocytes
Microglia
Neurons
Oligodendrocytes
OPCs
p21+ cells
D
Microglia
p16
p21
Cxcl10
Ctnnb1
Serpine1
Cxcl12
Csf1
Tnf
Il1b
Icam1
Igfbp4
Jun
Cxcl2
Il2
Gem
Sema3f
Il1a
Ccl5
Ccl4
Plaur
Spp1
Angptl4
Il15
Iqgap2
Igf1
Igfbp2
E
Oligodendrocytes
p16
p21
Inha
Egfr
Igf1
Vgf
Mmp12
Jun
Tnf
Il2
Fgf2
Vegfa
Ccl5
Igfbp2
Tnfrsf1a
Cd9
Il1a
Plat
Icam1
F
none
p16+
p21+
umap2
umap1
G
pseudotime
15
10
5
0
umap2
umap1

 © The Author(s)

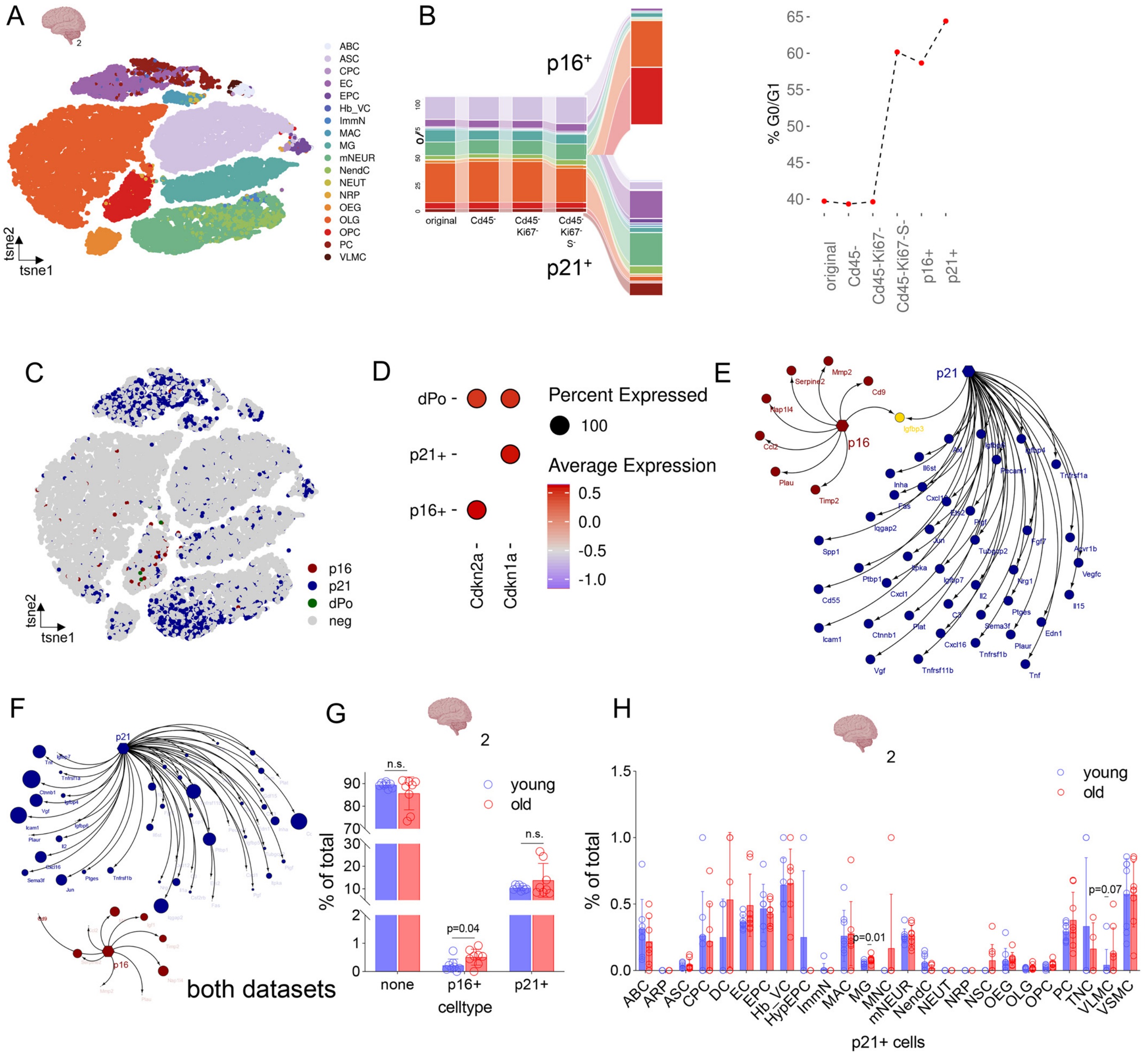

Figure EV2. A second brain sample (Ximerakis et al, 2019) was used to confirm the findings from the first brain sample.

(**A**) A t-SNE plot indicating all cell types. (**B**) The selection of *p16+* cells focuses on oligodendrocyte precursors, while *p21+* cells were mostly endothelial cells. (**C**) The t-SNE plot demonstrates that most cells are either *p16+* or *p21+*, but double-positive cells are rare. (**D**) The expression of *p16+*, *p21+* cells, and the rare dPo population. (**E**) There is only a very small overlap between the *p16*- and *p21*-positive secretory phenotype, namely just one gene (*Igfbp3*). (**F**) When combining both brain datasets, we observed no consistent overlap between the secretory profiles of *p16+* and *p21+* cells, underscoring that they represent distinct populations. Shared genes across both datasets are shown in solid color, whereas genes unique to a single dataset are displayed transparently. (**G**) *p16+* cells are significantly more frequent in the old brain. (**H**) Microglia (MG) has more *p21+* cells in the old brain. (**G**): unpaired *t*-test, *n* = 8 per condition, error bars: sd, (**H**) multiple paired *t*-tests, *n* = 8 per condition, error bars: sd.

© The Author(s)

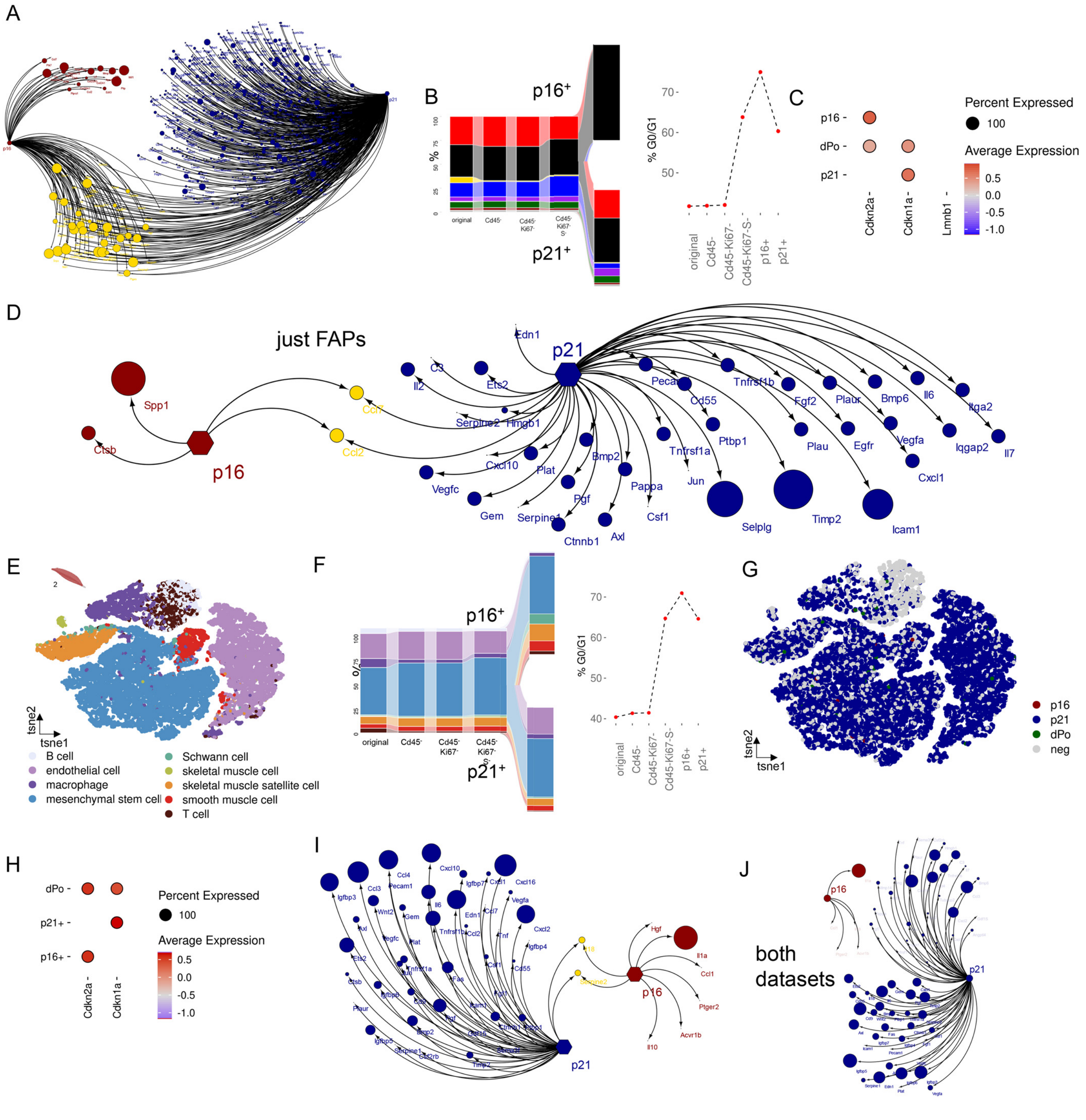

Figure EV3. Comprehensive analysis of mRNA expression profiles in aging murine muscle.

(**A**) In the muscle, there is some overlap with a noticeably larger p21-associated secretome compared to p16. (**B**) The selection criteria for *p21+* and *p16+* cells. While most of the *p16+* cells are FAPs (black), in the *p21+* cells there are also some endothelial cells (red). (**C**) Key markers for the abovementioned populations. (**D**) Secretory phenotype composition in *p16+* and *p21+* FAPs. (**E**) A second muscle dataset (Tabula_Muris_Consortium, 2020) showed nine key cell populations, with (**F**) most of the *p16+* and *p21+* cells originating from the mesenchymal stem cell compartment. (**G**) The majority of cells is *p21+*. (**H**) Key markers for these three populations. (**I**) The secreted factors between these two populations are heterogeneous, and (**J**) Integration of both datasets shows no overlap in secreted factors between these two populations.

 © The Author(s)

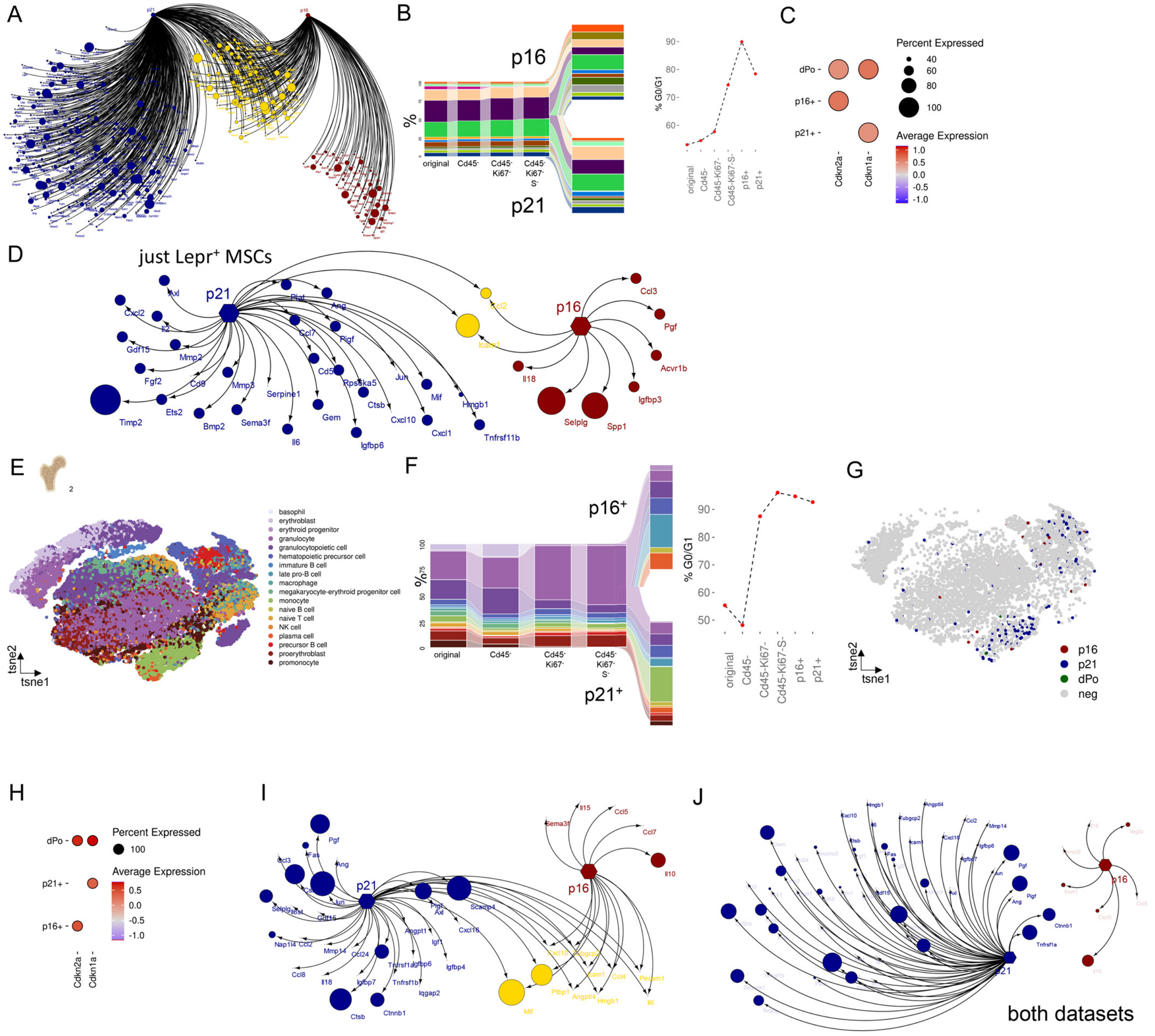

Figure EV4. Analysis of the secretome in bone during aging.

(**A**) Similar to the muscle, in the bone, there is some overlap but a more pronounced p21-associated secretome. (**B**) The selection criteria and key populations (**C**) for *p16*+ and *p21*+ cells. (**D**) Just Lepr+ MSCs and p16- and p21-associated SenMayo phenotype, respectively. (**E**) A second bone dataset (Tabula_Muris_Consortium, 2020) shows a (**F**) high amount of B cells in *p16*+ cells and monocytes in *p21*+ cells. (**G**, **H**) There are a few *p21*+ cells, and almost no dPo cells. (**I**) There is some overlap between *p16*+ and *p21*+ cells, while a summary of both bone datasets (**J**) shows no single gene overlapping for *p16*- and *p21*-associated secretory factors.

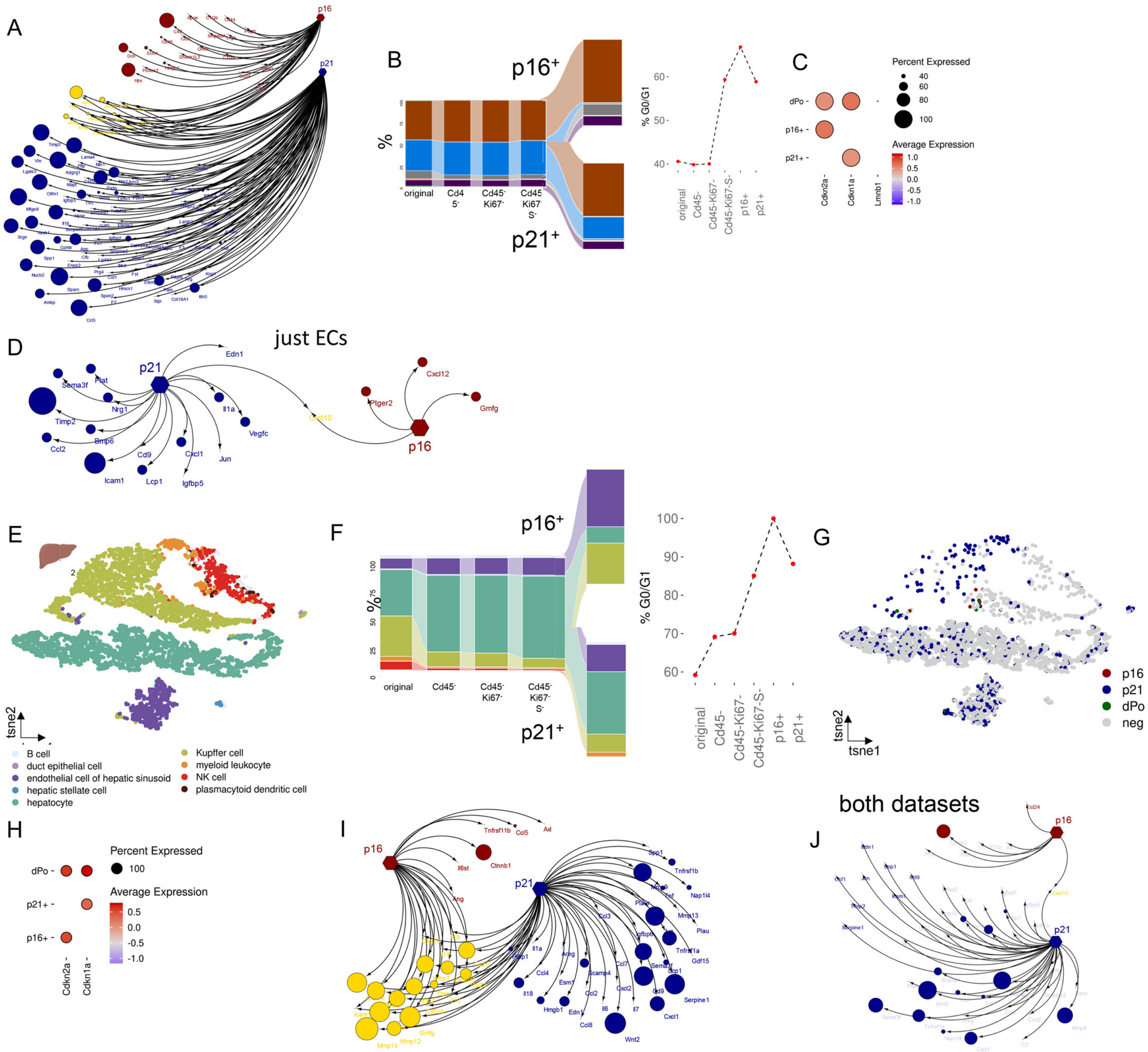

Figure EV5. Liver analysis demonstrates differences between *p21*+ cells and *p16*+ cells in aging.

(**A**) Within the liver, there is minimal overlap between *p21*+ cells and *p16*+ cells, with the *p21*+ cells predominantly expressing the majority of factors. (**B**) The selection of *p16*+ cells shows mostly endothelial cells, while in the *p21*+ cells, there are also some hepatocytes. (**C**) The key markers for the three key populations. (**D**) *p21*- and *p16*-associated SenMayo secretory phenotype in ECs. (**E**) A second hepatic dataset (Tabula_Muris_Consortium, 2020) was analyzed, while (**F**) endothelial cells were the most abundant in *p16*+ cells, and hepatocytes the most prominent in the *p21*+ cells. (**G**, **H**) *p21*+ cells were the majority compared to *p16*+ cells, and (**I**) there was a substantial overlap between *p21*- and *p16*-associated secretory factors, although *p21*+ cells expressed a greater number of factors than *p16*+ cells. (**J**) Combining both datasets, there was just one overlapping factor (*Cxcl16*).

 © The Author(s)

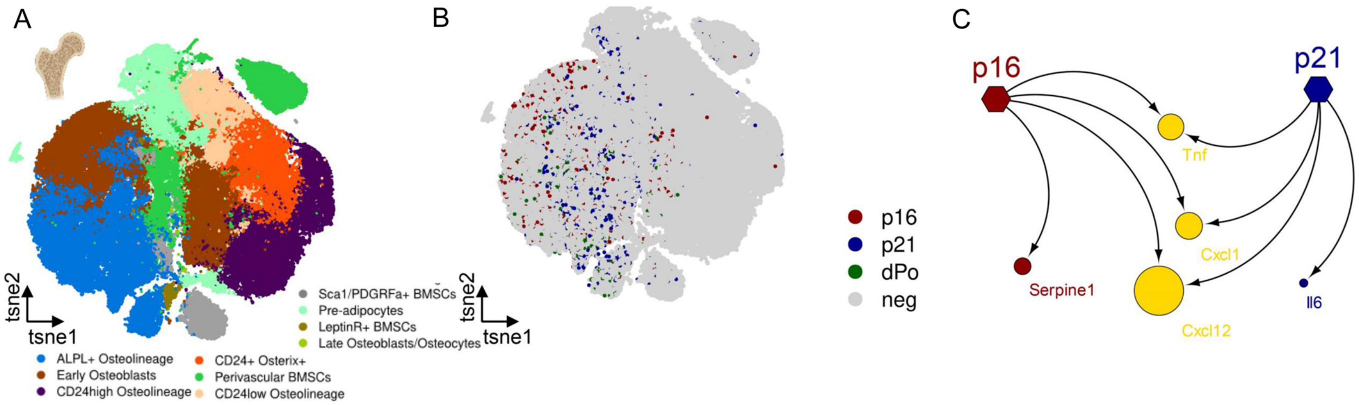

Figure EV6. CyTOF analyses of *p16*, *p21* and associated secreted factors in murine bone.

(**A**) CyTOF dataset from murine bone (Doolittle et al, 2023). (**B**) *p16*+ and *p21*+ cells are largely distinct, with minimal overlap. (**C**) The associated secretory phenotype of *p16*+ and *p21*+ cells shows some overlap but is limited to only a few factors, likely reflecting the limited antibody panel available.

© The Author(s)

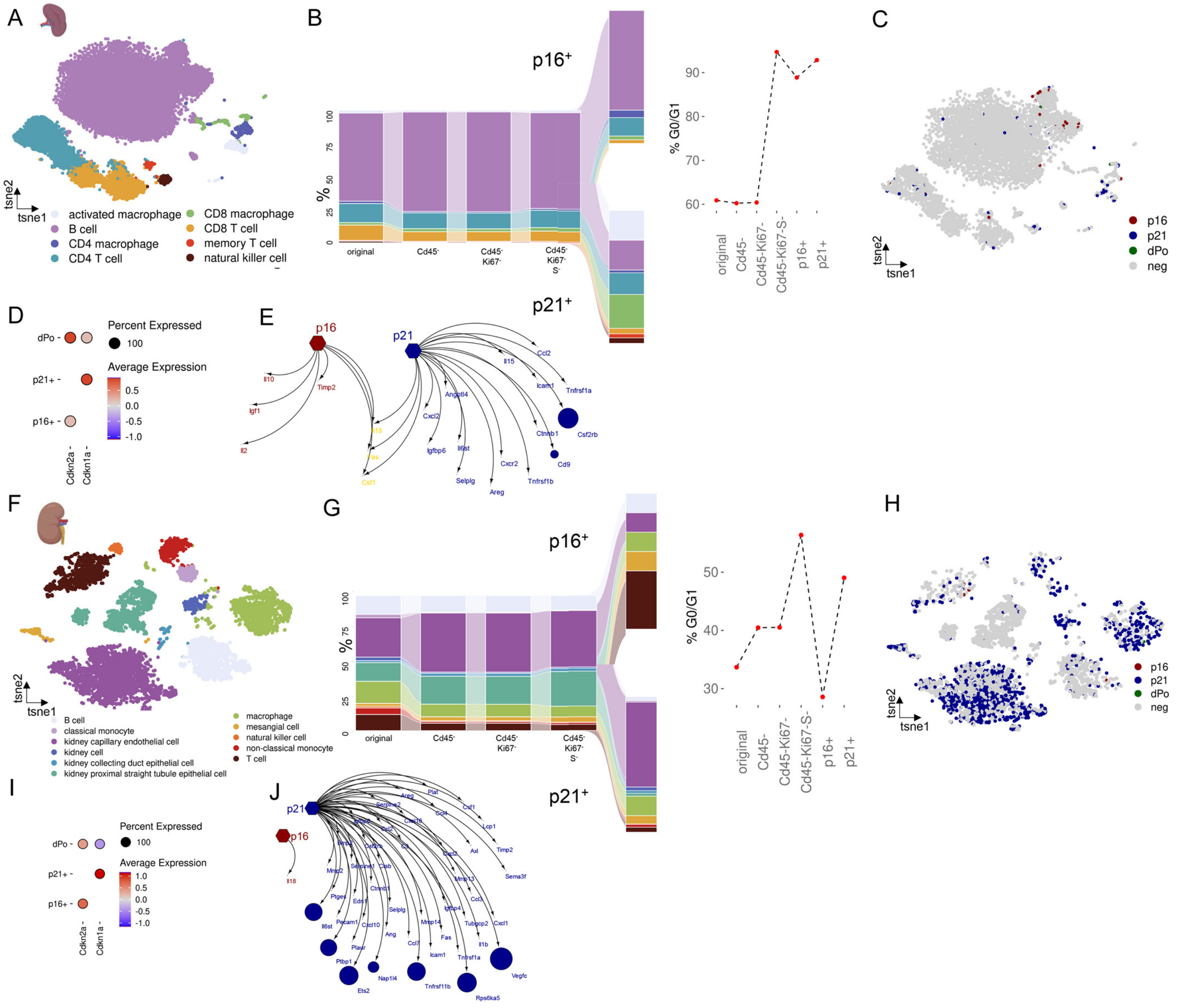

Figure EV7. Spleen and kidney display p16- and p21-associated differences consistent with previous murine datasets.

(**A**) In the spleen calico dataset (Kimmel et al, 2019, GSE 132901), eight different populations were identified and (**B**) analyzed according to their Cd45-negativity, Ki67-negativity and S-phase exclusion, followed by *p16*- (mostly B cells) and *p21*-positivity (mostly Cd8 macrophages). (**C**) The majority of cells were *p21+*, although (**D**) only a small fraction of total cells were positive for either marker. (**E**) Secreted factors showed limited overlap, with most associated with *p21+* cells. (**F**) The kidney dataset consisted of 11 different cell types, out of which (**G**) T cells were the most abundant in *p16+* cells and capillary endothelial cells were the most abundant in *p21+* cells. (**H, I**) The majority of cells was *p21+*. (**j**) A vast majority of secreted factors was associated with *p21+* cells.

 © The Author(s)

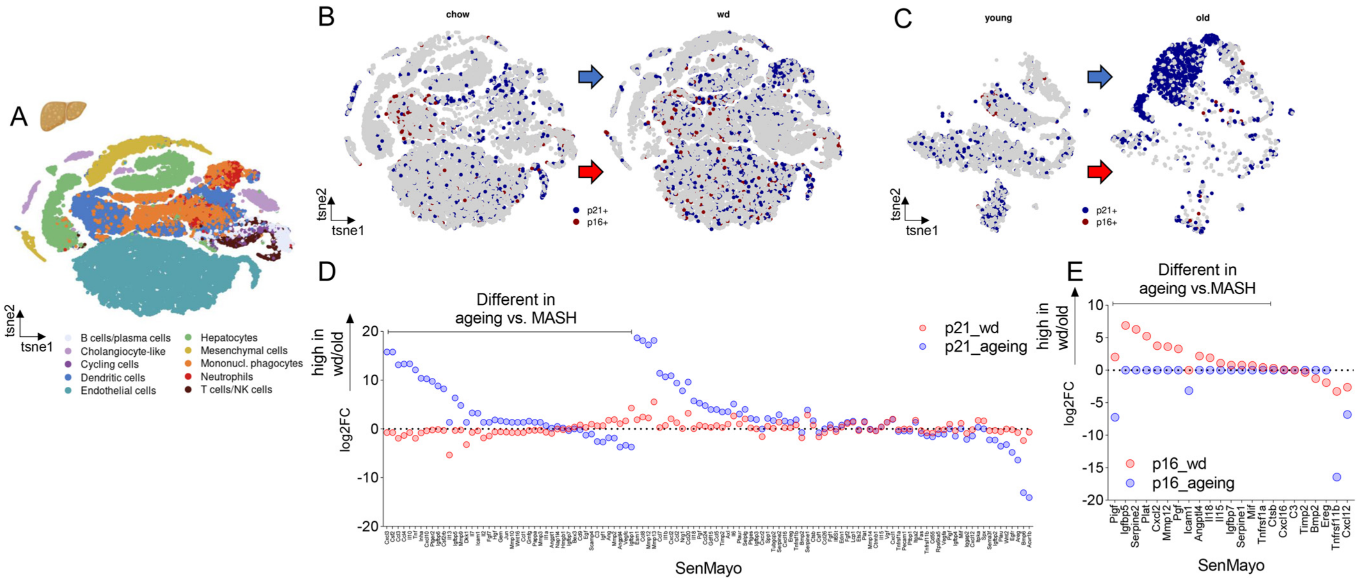

Figure EV8. Distinct secretory phenotypes of *p16*+ and *p21*+ cells in MASH and aging liver.

(**A**) Analysis of a diseased liver (MASH, GSE218300, (Bendixen et al, 2024)), (**B**) a western-style diet (wd) compared to chow resulted in a moderate increase of *p21*+ cells, while (**C**) aging resulted in a profound increase of *p21*+ cells. (**D**) The secretory phenotype in *p21*+ cells was different to a large degree in aging vs. MASH, with a higher amount of genes upregulated in aging. Similarly, the (**E**) secretory phenotype in *p16*+ cells was markedly different in aging vs. MASH, but with more genes upregulated in the western diet compared to aging.

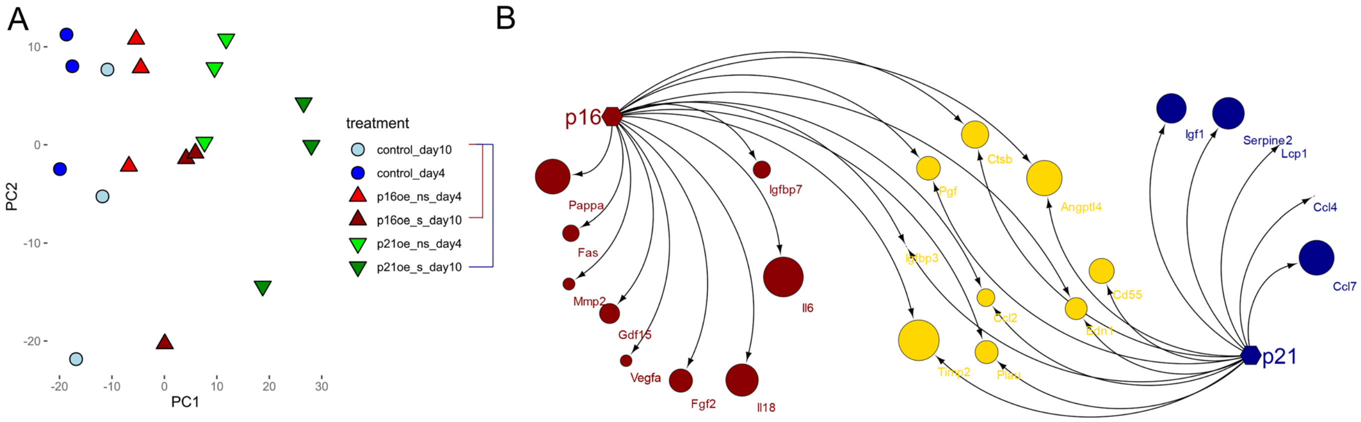

Figure EV9. Overexpression of p16 and p21 reveals distinct secretory profiles (GSE117278).

(**A**) A PCA plot illustrating control-MEFs on day 4 and 10, as well as p16-overexpressing cells (adeno-Cre-EGFP virus Ai14;L-p16 injection into the tail) on day 4 and 10, compared to p21-overexpressing cells (Ai14;L-p21) on day 4 and 10. (**B**) A comparable number of factors are expressed in p16+ (red)- vs. p21+ (blue) cells, with overlap indicated in yellow.

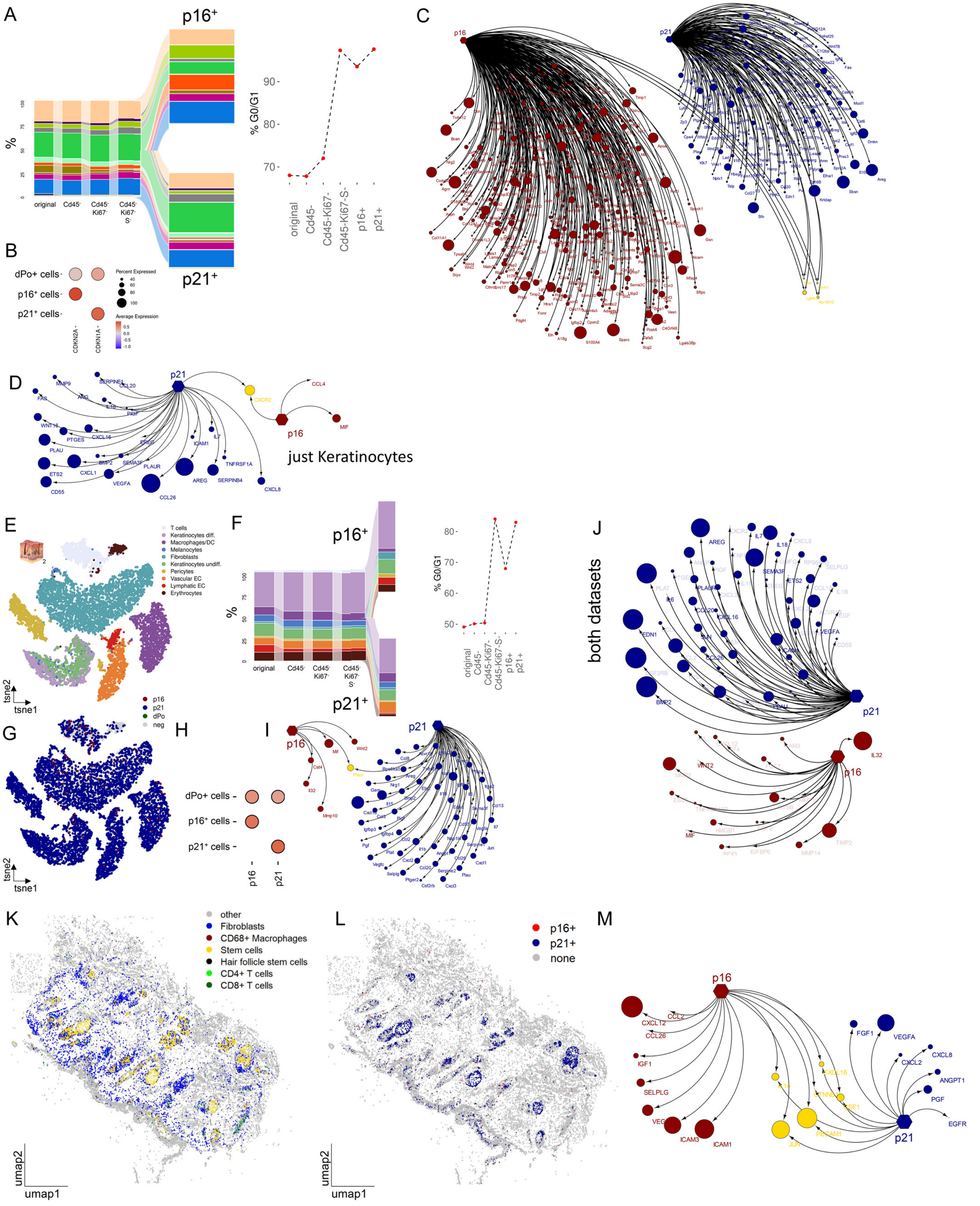

A
p16⁺
p21⁺
%
original
Cd45⁻
Cd45⁻ Ki67⁻
Cd45⁻ Ki67⁻ S⁻
% G0/G1
B
dPo+ cells
p16⁺ cells
p21⁺ cells
CDKN2A
CDKN1A
Percent Expressed
Average Expression
C
p16
p21
D
just Keratinocytes
E
F
G
H
I
J
both datasets
K
other
Fibroblasts
CD68+ Macrophages
Stem cells
Hair follicle stem cells
CD4+ T cells
CD8+ T cells
umap1
umap2
L
p16+
p21+
none
M

Figure EV10. Human skin secretome, associated with p21 and p16, incorporating datasets from Zou et al, (2021) and Solé-Boldo et al, (2020).

(**A**) In the skin, there is minimal overlap between *p16+* and *p21+* cells in terms of mRNA expression of the whole secretome. Unlike the majority of examined tissues, the mRNA expression of the entire secretome associated with p16 is as extensive as that associated with p21. (**B**) CD45-negativity, followed by Ki67-negativity and exclusion of S-phase cells were followed by p21- or p16-positivity for selection of proposedly senescent cells, which key markers are depicted in (**C**). (**D**) SenMayo in *p16+* and *p21+* keratinocytes, respectively. (**E**) Five human skin samples (GSE130973) were likewise (**F**) applied these selection criteria, but *p16+* cells were lacking, resulting in an abundance of (**G**) *p21+* cells and (**H**) no *p16+* cells. (**I**) Subsequently, there is just a p21-associated SASP, and no overlap (**J**) in both datasets for *p16+* and *p21+* cells. (**K**) Multiplexed error-robust fluorescence in situ hybridization (MERFISH) analysis on human skin samples, with different cell types within a human skin sample. Within these cell types, (**L**) *p16+* (red) and *p21+* (blue) cells are largely distinct, with minimal overlap between these markers. (**M**) *p16+* and *p21+*-associated secretory profiles within the MERFISH dataset.

 © The Author(s)

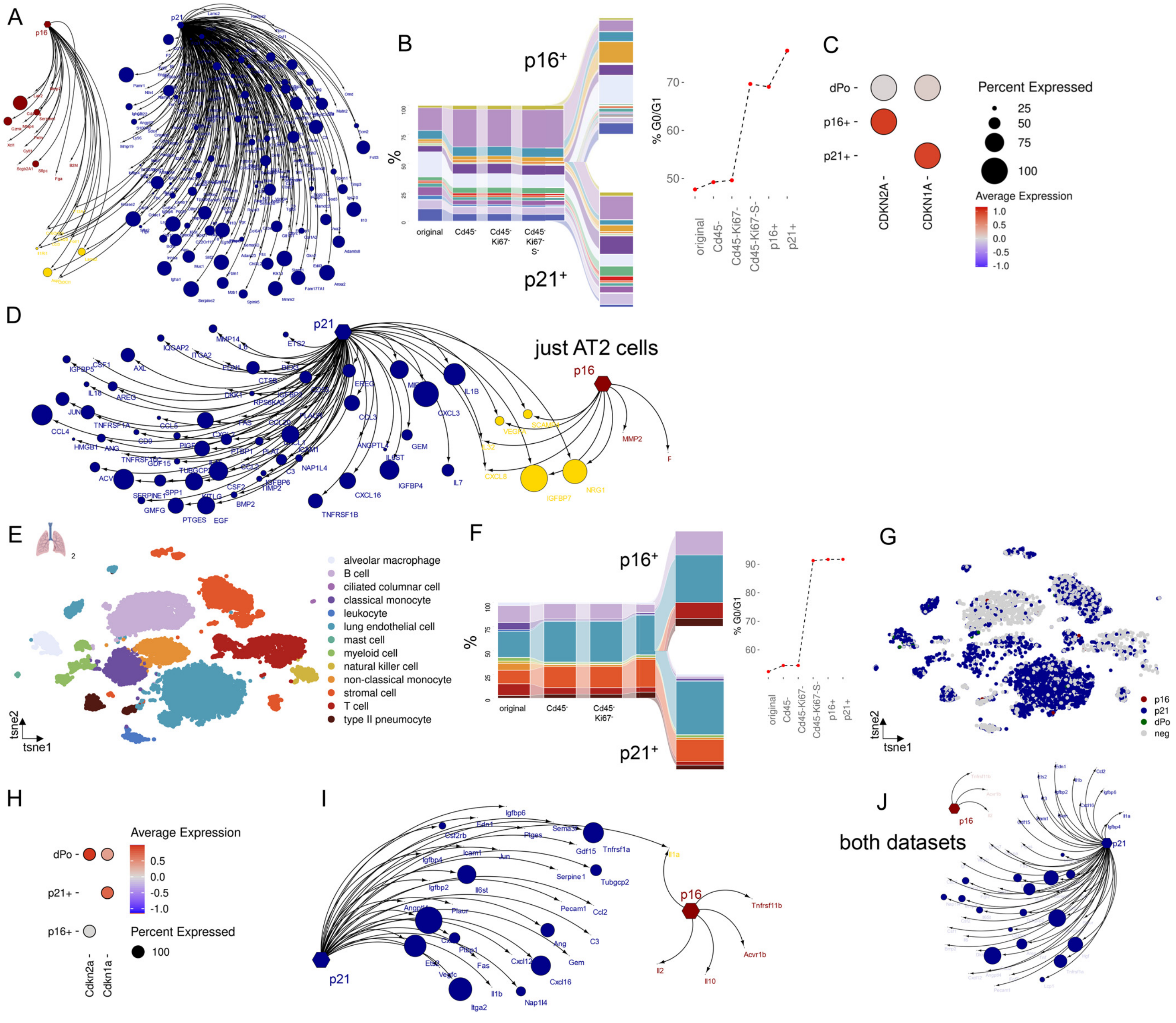

Figure EV11. Analysis of lung reveals limited overlap in the secretory phenotype and distinct developmental profiles of p16- and p21-positive cells.

(**A**) In the human lung, the *p21*+ cells express a large number of secreted factors, with a very small overlap with *p16*+ cells. (**B**) Selection criteria for human lung tissue include CD45-negativity, followed by Ki67-negativity and exclusion of S-phase cells, before the cells are distributed between p16+ or p21+. (**C**) The markers for these three key populations. (**D**) *p16*- and *p21*-SenMayo associated secretory phenotype in *p16*+ and *p21*+ AT2 cells, respectively. (**E**) The murine Calico lung dataset (Kimmel et al, 2019) provides 13 different cell types, which are selected (**F**) as abovementioned. (**G**, **H**) The vast majority of cells are *p21*+. (**I**) The secretory phenotype itself is more abundant in the *p21*+ cells and there is (**I**) a small overlap of *p16*+ and *p21*+ associated factors between both datasets.

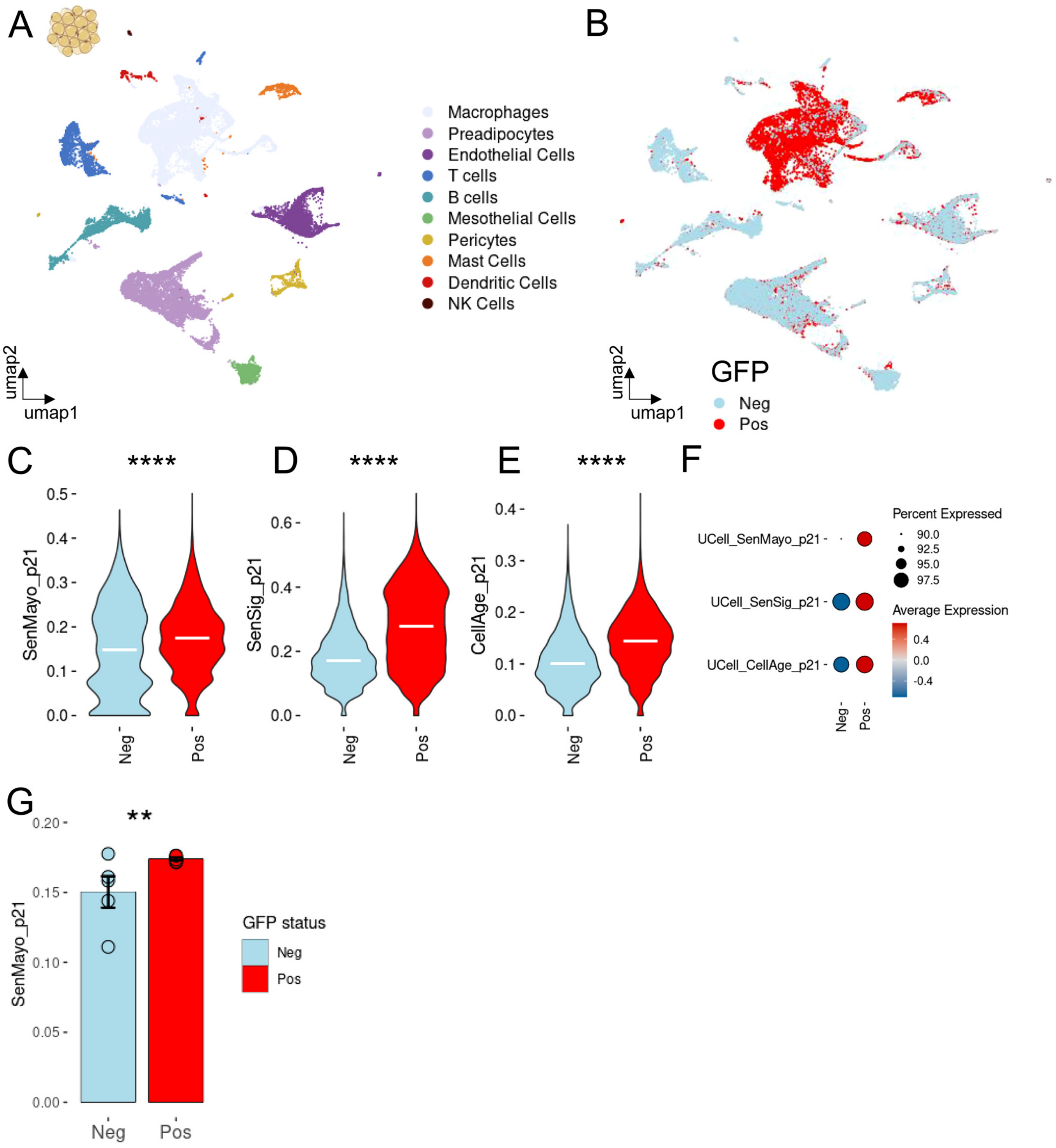

Figure EV12. In an adipose tissue dataset (Wang et al, 2024a, GSE269660), the GFP/Cdkn1a-positive cells exhibited an enrichment of the p21-associated secretory phenotype.

(**A**) In adipose tissue, ten different populations were identified, out of which the (**B**) macrophages were the highest in *p21/GFP*. (**C**) The *p21*-associated SenMayo, (**D**) SenSig, and (**E**) CellAge were all (**F**) significantly higher in the *GFP*-positive cells compared to the *GFP*-negative cells. (**G**) Likewise, a samplewise comparison ($n = 10$) between *GFP*-positive ($n = 5$) and *GFP*-negative ($n = 5$) samples shows an increase of *p21*-associated SenMayo in the positive samples. *****p* value <0.0001, ****p* value <0.001, ***p* value <0.01, **p* value <0.05. (**C–E**) Exact *p* values $= 2 \times 10^{-16}$, Wilcoxon test, (**G**) Exact *p* value = 0.004, *t*-test, error bars: 2 SE. $n = 5$ per condition

 © The Author(s)

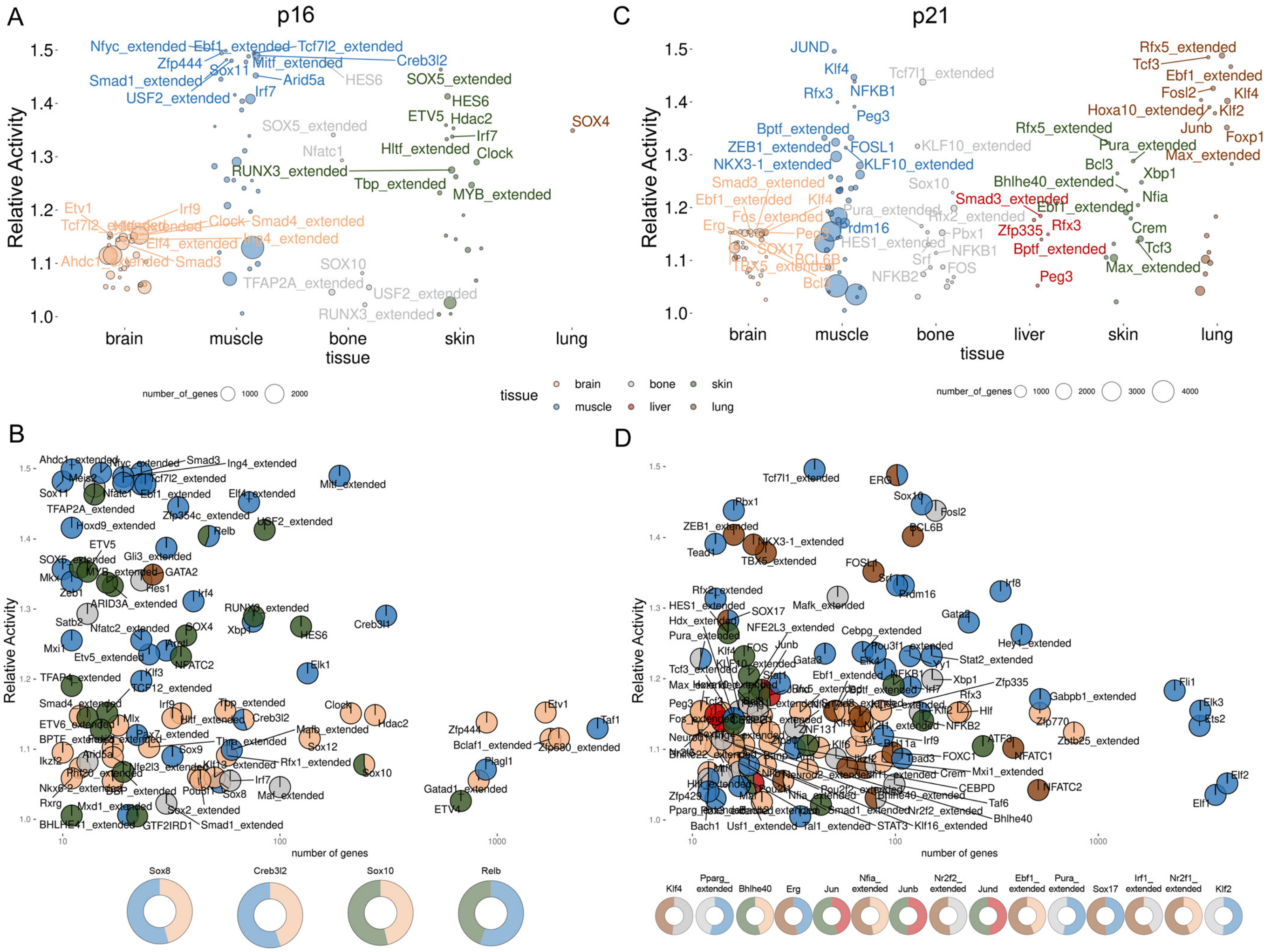

Figure EV13. SCENIC analysis of the regulating transcription factors of *p16*+ and *p21*+ cells.

(**A**) When analyzing five distinct tissues for regulatory factors in *p16*+ cells, each tissue exhibits a substantial array of transcription factors governing the behavior of *p16*+ cells. (Since the liver has very few p16-positive cells for proper calculation with SCENIC, these were excluded). (**B**) The x-axis represents the number of genes, plotted against the y-axis, illustrating the relative activity of the respective transcription factor, highlighting the significance of each factor. Interestingly, only four factors (*Sox8*, *Creb3l2*, *Sox10*, and *Relb*) exhibit consistency across multiple tissues in the context of *p16*+ cells. (**C**) In *p21*+ cells, the regulating transcription factors show a high heterogeneity across tissues. (**D**) The transcription factors associated with *p21* are predominantly specific to a single organ, but a few transcription factors, such as *Erg*, *Sox17*, *Klf4*, *Jun*, *Klf2*, and others, regulate *p21*+ cells in two tissues.

© The Author(s)
